# Artificial Intelligence in Pancreatic Endoscopic Ultrasonography: From Image-Based Diagnosis to Cytopathology

**DOI:** 10.3390/jcm15187167

**Published:** 2026-09-15

**Authors:** Elettra Merola, Leonardo Sosa Valencia, Nico Pagano, Maria Pina Dore, Julieta Montanelli, Claudio De Angelis, Abdenor Badaoui

**Affiliations:** 1 Department of Medicine, Surgery and Pharmacy, University of Sassari, 07100 Sassari, Italy; mpdore@uniss.it; 2 IHU-Strasbourg, Institute of Image-Guided Surgery, Université de Strasbourg, 67000 Strasbourg, France; leonardo.sosa-valencia@ihu-strasbourg.eu (L.S.V.); julieta.montanelli@ihu-strasbourg.eu (J.M.); abdenor.badaoui@chuuclnamur.uclouvain.be (A.B.); 3 Unit of Gastroenterology, Ospedale Maggiore della Carità di Novara, 28100 Novara, Italy; nicopagano@gmail.com; 4 Gastroenterology and Endoscopy Department, Koelliker Hospital, Corso Galileo Ferraris, 247/255, 10134 Torino, Italy; eusdeang@gmail.com; 5 Department of Gastroenterology and Hepatology, CHU UCL Namur, Université Catholique de Louvain, 5530 Yvoir, Belgium; 6 Institute for Translational Medicine and Liver Disease, ITM, UMRS_1110, Université de Strasbourg, 67000 Strasbourg, France; 7 Department of Gastroenterology and Hepatology, Hôpitaux Universitaires de Strasbourg, Université de Strasbourg, 67000 Strasbourg, France

**Keywords:** artificial intelligence, endoscopic ultrasonography, pancreatic diseases, EUS-guided tissue acquisition, cytopathology

## Abstract

The use of artificial intelligence (AI) in endoscopic ultrasonography (EUS) is receiving increasing attention, particularly in the field of pancreatic diseases, where early and accurate diagnosis remains a major clinical challenge. This narrative review focuses on current and emerging applications of AI in pancreatic EUS, covering both image-based diagnostic support and the analysis of samples obtained through EUS-guided tissue acquisition. The first part of the review discusses how AI is being applied to improve EUS image interpretation, ranging from lesion detection to characterization and differentiation between benign and malignant pancreatic findings. The review also discusses early evidence and future perspectives for real-time procedural support, where diagnostic performance remains highly operator-dependent. The second part explores a less frequently discussed but equally relevant area: the use of AI in the analysis of cytological specimens obtained through EUS-guided fine-needle aspiration or fine-needle biopsy. Although cytopathology may appear to lie outside the traditional clinical scope of EUS, it represents an essential step in the diagnostic workflow of pancreatic diseases. Recent developments in AI-assisted digital cytology and pathology have shown promising potential to support and standardize cytological interpretation, with possible benefits in terms of diagnostic consistency, reproducibility, and turnaround time. By bridging imaging and pathology, AI may enhance the entire pancreatic EUS workflow, contributing to more efficient, accurate, and personalized diagnostic pathways in pancreatic disease management.

## 1. Introduction

Endoscopic ultrasonography (EUS) has become a key component of the diagnostic pathway for pancreatic diseases, as it combines high-resolution imaging of the pancreatic parenchyma with the possibility of obtaining cytological or histological samples through EUS-guided tissue acquisition. The technical evolution of EUS-guided sampling, including the increasing use of fine-needle biopsy (FNB) needles and standardized sampling strategies, further reflects the central role of EUS in the modern multidisciplinary management of pancreatic lesions [1].

Despite these strengths, pancreatic EUS remains a complex and operator-dependent procedure. Image interpretation may be influenced by endosonographer experience, image quality, lesion size and location, and the presence of background pancreatic changes. In daily practice, differentiating pancreatic ductal adenocarcinoma (PDAC) from chronic pancreatitis, pancreatic neuroendocrine tumors (PanNETs), autoimmune pancreatitis (AIP), metastases, or other less common pancreatic lesions can be challenging when imaging features overlap. This distinction is clinically relevant because these entities require different therapeutic strategies and have markedly different prognostic implications. Moreover, even when tissue acquisition is performed, diagnostic yield may be affected by needle type, sampling technique, number of passes, specimen handling, and the availability of rapid on-site evaluation (ROSE) [2].

Artificial intelligence (AI) has emerged as a promising approach to address some of these limitations. Most available evidence has focused on image-based diagnosis. AI models have been investigated for pancreatic tumor detection, characterization of solid masses, and differentiation between specific tumor types. Recent approaches have also incorporated nomograms to support differential diagnosis and improve clinical interpretability [3]. In pancreatic EUS, AI may support image interpretation by extracting visual patterns that are not immediately apparent to the human eye, assisting lesion detection, and improving differentiation between benign and malignant findings. Deep learning (DL), particularly convolutional neural network (CNN)-based approaches, has been widely explored because of its ability to learn complex image features directly from EUS images or videos. A recent systematic review and meta-analysis of DL-assisted EUS for pancreatic tumors reported high pooled diagnostic performance, suggesting its potential role as a computer-aided diagnostic support tool; however, the authors also emphasized the need for further validation before clinical implementation [4]. These applications are clinically meaningful because distinguishing PDAC from PanNETs may substantially influence therapeutic decision-making, surgical planning, and prognosis [5]. Furthermore, AI has recently been applied to EUS-based prediction of PanNET grading, with encouraging preliminary results [6].

However, the diagnostic workflow of pancreatic EUS does not end with image interpretation. EUS-guided tissue acquisition and subsequent cytological or histological assessment remain essential, especially when pathological confirmation is required before oncological treatment. Current technical guidance highlights the importance of specimen adequacy, tissue integrity, and appropriate processing [2]. Therefore, a comprehensive discussion of AI in pancreatic EUS should not be limited to real-time image analysis but should also consider how AI may assist the downstream evaluation of samples obtained during EUS-guided procedures.

AI-assisted digital cytology represents a less frequently discussed but increasingly relevant area in this field. Digital cytology and whole-slide imaging (WSI) are creating new opportunities for automated or semi-automated assessment of cytological specimens, including cell detection, adequacy assessment, classification support, and workflow prioritization. Although AI applications in cytology have a longer history in cervical screening, recent expert reviews have highlighted growing interest in extending AI-based approaches to non-gynecological cytology, including fine-needle aspiration (FNA) specimens and pancreatobiliary samples. A recent meta-analysis showed that computer-assisted diagnosis (CAD) in pancreatic cytology can achieve near-expert accuracy for definitive diagnoses, potentially reducing the burden of routine screening and accelerating surgical planning [7]. Nevertheless, many of these applications remain at the research or early validation stage. By bridging imaging and pathology, AI may contribute to a more standardized, efficient, and personalized diagnostic pathway for patients with pancreatic diseases, provided that future studies adequately address external validation, clinical integration, explainability, data governance, and real-world impact on patient management.

The rationale for this narrative review arises from this evolving and fragmented landscape. Existing literature has mainly addressed AI in EUS image interpretation, whereas the connection between EUS imaging, tissue acquisition, and cytopathology has received less attention. The available evidence is heterogeneous, including proof-of-concept studies, retrospective model-development cohorts, meta-analyses of diagnostic accuracy, technical guidance on EUS-guided tissue acquisition, and broader digital cytology literature. Rather than providing a pooled estimate of diagnostic performance, this review aims to place these data into a clinically meaningful framework, providing an updated overview focusing on two complementary domains: image-based diagnostic support and AI-assisted analysis of samples obtained through EUS-guided tissue acquisition.

## 2. Methods

An updated structured narrative literature search was performed to identify original studies investigating the application of AI techniques to pancreatic EUS. The search strategy combined terms related to EUS and AI, using the following query: (EUS OR “endoscopic ultrasonography”) AND (AI OR “artificial intelligence” OR “machine learning” OR “deep learning”). The search was restricted to articles published over the previous five years, from 1 January 2021 to 25 May 2026. This time frame was selected to focus on contemporary AI architectures, current EUS technology, tissue-acquisition workflows, digital cytology/pathology applications, and validation standards relevant to present clinical practice. PubMed was selected as the primary bibliographic source because the review was intended as a clinically oriented narrative synthesis focused on pancreatic EUS, EUS-guided tissue acquisition, cytology, and pathology, rather than as an exhaustive technical review of all AI and image-processing literature.

Overall, the PubMed search retrieved 755 records. Eligibility criteria are summarized in Supplementary Table S1. Studies were considered eligible if they reported original data on AI-based models applied to EUS imaging or EUS-guided cytological/histological assessment for the diagnosis of pancreatic diseases. Articles not focused on pancreatic diseases, review articles, meta-analyses, perspectives, guidelines, correspondence, conference abstracts, non-peer-reviewed material, and studies in which AI was not a core analytical component were excluded.

Because the search was conducted in a single bibliographic database, no formal multi-database duplicate removal workflow was required. When articles already retrieved through the PubMed search were also identified during manual reference checking, they were not counted twice. To improve completeness, the reference lists of eligible articles were manually reviewed to identify additional relevant original publications that fulfilled the inclusion criteria but had not been captured by the initial PubMed search.

Article selection was performed by two authors (E.M. and M.P.D.) according to the predefined eligibility criteria reported in Supplementary Table S1. Data from included studies were extracted into structured summary tables including study design, dataset characteristics, AI method, diagnostic task, main findings, and main limitations. Any uncertainties regarding study eligibility or data interpretation were discussed among the authors until consensus was reached.

Given the narrative design of the review and the heterogeneity of the included studies, no formal risk-of-bias score or quality-assessment tool was applied. However, a structured qualitative appraisal of AI-relevant methodological features was performed. Key domains included study design, setting, sample size, type of input data, image-level versus patient-level analysis or splitting when reported, validation approach, external validation when available, reference standard, and whether the model was tested on still images, videos, or in a real-time/prospective setting. These features were extracted when reported, summarized in the tables, and discussed throughout the manuscript.

Overall, fifty studies met the inclusion criteria and were included in the present narrative review.

AI-based tools were used to support language editing, stylistic refinement, and figure preparation (ChatGPT-5.5, OpenAI). All decisions regarding study selection, data interpretation, manuscript content, and scientific conclusions were made by the authors, who remain fully responsible for the integrity and accuracy of the work.

## 3. AI Applications for Image-Based Diagnosis in Pancreatic EUS

Image-based diagnosis represents the most extensively explored application of AI in pancreatic EUS, with models developed to assist lesion detection, characterization, differential diagnosis, risk stratification, anatomical recognition, procedural guidance, and examination quality throughout the EUS workflow. Detailed study-level information is provided in Supplementary Tables S2–S5. In the following sections, the main text focuses on the principal clinical directions, comparative interpretation, and methodological issues most relevant to clinical translation.

### 3.1. Pancreatic Lesion Detection and Quality Control

Lesion detection and quality control represent clinically intuitive applications of AI in pancreatic EUS, as they may support the endosonographer in identifying suspicious abnormalities, improving completeness of examination, and reducing operator dependence. Overall, six studies addressing this topic were identified and are summarized in Supplementary Table S2 [8,9,10,11,12,13].

Two main directions emerge from this literature. First, AI has been applied as a real-time or near-real-time quality-control tool during biliopancreatic EUS. Li et al. [8] developed “EUS-AIRS”, a DL-based automatic image-reporting system that improved completeness of standard-station documentation compared with endoscopists, although its main focus was procedural documentation rather than diagnostic classification. Similarly, Wu et al. [9] prospectively validated EUS-IREAD in 290 randomized patients, showing a reduction in missed standard stations and anatomical structures without increasing procedure time. These studies suggest that one of the most mature short-term applications of AI in pancreaticobiliary EUS may be workflow support and quality control rather than autonomous diagnosis.

Second, AI models have been developed for image- or video-based lesion characterization across different EUS modalities. Podină et al. [10] evaluated AI-assisted interpretation of contrast-enhanced (CE-)EUS videos in a prospective multicenter observer-blinded study and showed high performance for a dedicated AI system, whereas ChatGPT Agent Mode performed poorly and was included only as an exploratory general-purpose comparator. Zhang et al. [11], Saraiva et al. [12], and Udriștoiu et al. [13] further explored multimodal, CNN-based, and hybrid approaches for differentiating solid, cystic, and non-cancerous pancreatic lesions, often reporting high diagnostic accuracy. However, these findings should be interpreted cautiously because several studies relied on retrospective datasets, selected still images or videos, limited lesion spectra, unclear patient-level separation, augmentation, or lack of broad prospective multicenter validation.

### 3.2. Differential Diagnosis of Solid Pancreatic Lesions: PDAC Diagnosis

Given the central role of EUS in the evaluation of solid pancreatic lesions, several AI-based models have been developed to support the differential diagnosis of PDAC from benign, inflammatory, or other neoplastic pancreatic conditions. These studies are summarized in Supplementary Table S3 [14,15,16,17,18,19,20,21,22]. Overall, the available evidence includes retrospective image- or video-based classifiers, prospective validation cohorts, and reader-assistance studies.

#### 3.2.1. Retrospective Studies

Retrospective studies have mainly explored the feasibility of AI-assisted PDAC detection or differentiation from non-carcinomatous and inflammatory pancreatic lesions. Ruano et al. [14] evaluated a machine learning (ML) approach intended to analyze the full EUS examination rather than selected representative images, supporting the concept of procedure-level computational assessment but remaining limited by the use of handcrafted features and a relatively small cohort. Kuwahara et al. [15] subsequently applied an EfficientNetV2-L DL model to a larger single-center dataset with temporally separated testing, achieving good patient-level performance for PDAC detection.

Other studies moved toward real-time or externally validated image-based detection. Tian et al. [16] developed a YOLOv5m-based real-time auxiliary diagnosis, although its performance was not significantly superior to physician EUS assessment without puncture. Hu et al. [17] evaluated a modified Faster R-CNN model with external image validation and showed that AI assistance could improve physicians’ diagnostic performance.

A specific subgroup of studies addressed the clinically challenging distinction between AIP and PDAC. Marya et al. [18] used a CNN model trained on still images and videos and reported high performance in differentiating AIP from PDAC, while Nakamura et al. [19] applied ResNet152 to quasi-RGB images generated from EUS video frames, generally outperforming endoscopists. Taken together, these retrospective studies suggest that AI may support PDAC detection and differential diagnosis, particularly in visually ambiguous cases. However, most evidence still derives from single-center or image-based settings, and prospective external validation in real-time EUS practice remains limited.

#### 3.2.2. Prospective Studies

Two studies published in the last five years have prospectively explored the potential role of AI-assisted EUS in supporting the differential diagnosis of PDAC from other pancreatic conditions [20,21]. Gu et al. [20] developed a ResNet50-based DL radiomics model to differentiate PDAC from benign pancreatic lesions and prospectively tested its performance, showing good diagnostic accuracy and improved reader performance, particularly among junior endosonographers. Cui et al. [21] developed a multimodal joint-AI model integrating EUS images and clinical variables, with internal and external testing followed by a prospective randomized crossover reader trial. This model achieved high diagnostic performance and improved novice interpretation, supporting the potential value of multimodal AI and human–AI collaboration. However, both studies were based on EUS images rather than live procedural deployment, and further real-world validation is needed before routine clinical implementation.

#### 3.2.3. Clinical Impact of AI Assistance on Endosonographer Performance

Beyond standalone algorithm performance, a key translational question is whether AI improves physician interpretation. Cui et al. [21] showed that AI assistance increased diagnostic performance mainly among less experienced endosonographers, whereas the incremental benefit for experts was less evident. This supports the concept of AI as a decision-support tool, training adjunct, or second observer rather than a replacement for expert assessment.

Building on this concept, Montanelli et al. [22] evaluated AI-assisted detection of small solid pancreatic tumors during dynamic EUS video interpretation. In this randomized tandem video reader study, AI assistance improved overall reader accuracy, particularly in a task designed to approximate the challenge of detecting small lesions during pancreatic screening. Although this study was based on video clips rather than live real-time EUS procedures, it represents an important step toward clinically meaningful evaluation of human–AI collaboration.

Overall, prospective and reader-assistance studies suggest that AI may be most clinically useful when used to augment endosonographer performance, especially among less experienced operators or in difficult detection tasks. Future studies should increasingly prioritize real-time workflow integration, user interaction, and patient-centered endpoints rather than standalone diagnostic accuracy alone.

### 3.3. Differential Diagnosis of Solid Pancreatic Lesions: PanNET and Grading Definition

AI-assisted EUS has also been investigated in the assessment of PanNETs, where overlap in echographic appearance with other solid pancreatic lesions and functional heterogeneity may complicate conventional interpretation. Studies addressing PanNET diagnosis, functional characterization, and grading prediction are summarized in Supplementary Table S3 [3,23,24,25,26,27,28,29,30].

Yi et al. [3] developed an interpretable EUS-based DL model and nomogram to distinguish PanNETs from pancreatic cancer, showing promising performance but also a reduction between training and test performance. Across a series of retrospective studies, Mo et al. further explored EUS-based ultrasomics, radiomics, and DL approaches for PanNET characterization [23,24,25,26]. These studies progressively evaluated quantitative EUS-derived features for distinguishing PanNETs from pancreatic cancer, differentiating insulinomas from non-functional PanNETs, and integrating radiomics or DL signatures with clinical and semantic EUS variables. Overall, combined models and nomograms generally performed better than isolated imaging signatures, but several studies showed lower test-cohort than training-cohort performance, supporting cautious interpretation of the highest reported AUCs and suggesting possible overfitting.

From a methodological perspective, these radiomics and ultrasomics studies should be interpreted within the complete radiomics pipeline, which includes image acquisition, preprocessing, ROI segmentation, feature extraction, feature selection or dimensionality reduction, model development, and validation [31]. Each step may affect reproducibility and generalizability. In EUS, acquisition variability is particularly relevant because images may differ according to endoscope platform, processor, gain, depth, focal settings, scanning plane, operator technique, and the use of contrast enhancement or elastography. Preprocessing choices, including image normalization, resizing or resampling, gray-level discretization, and filtering, may further alter texture and higher-order features. ROI segmentation is another critical source of variability, especially when manual intratumoral or peritumoral contours are used, as small differences in lesion boundaries may substantially influence extracted features. Feature stability analysis and harmonization procedures may improve reproducibility but were not consistently reported across the available EUS studies. Similarly, feature selection methods such as LASSO or tree-based importance ranking may reduce dimensionality but can also introduce overfitting if not embedded within an appropriate training-validation framework. For these reasons, the high AUCs reported in some EUS-radiomics and ultrasomics studies should be interpreted cautiously when external validation, patient-level splitting, harmonization, calibration, or prospective testing are absent. This methodological framework is particularly important for PanNET studies, where small cohorts, manual ROI segmentation, heterogeneous EUS equipment, and limited external validation remain recurrent limitations.

Beyond lesion detection and differential diagnosis, EUS-based AI has also been explored for the preoperative prediction of PanNET grading, which represents one of the most important prognostic factors for NETs [32,33]. Kiremitci et al. [27] evaluated DL applied to EUS images for predicting pathological PanNET grade, reporting high overall and grade-specific accuracy, although the study was limited by its small cohort, image-level modeling, class imbalance, and use of SMOTE-generated samples. Mo et al. subsequently extended EUS-based ultrasomics and DL approaches to grading prediction [28,29,30]. These studies evaluated G1 versus G2/G3 classification using ultrasomics, intratumoral–peritumoral features, and interpretable DL signatures, with encouraging but heterogeneous performance. Notably, the most recent study included external centers as a test cohort, representing a step toward broader validation, although prospective evaluation remains lacking.

Overall, these studies suggest that quantitative EUS-derived features, particularly when combined with clinical variables, semantic EUS features, and explainability tools, may support PanNET diagnosis, functional characterization, and grading prediction. However, this field remains preliminary. At present, AI-based EUS models should be regarded as investigational adjuncts and cannot replace histology, Ki-67 assessment, or multidisciplinary evaluation.

### 3.4. AI for Pancreatic Cystic Lesions

Regarding the differential diagnosis of pancreatic cystic lesions (PCL), AI-guided EUS has been mainly explored for cyst-type differentiation and IPMN risk stratification. Seven studies published in the last five years addressed this topic and are summarized in Supplementary Table S4 [34,35,36,37,38,39,40].

Early studies focused on differentiating mucinous from non-mucinous or serous from mucinous cystic lesions. Vilas-Boas et al. [34] developed an Xception-based CNN to distinguish mucinous from non-mucinous PCL using EUS video frames, reporting very high performance in a small retrospective pilot dataset. Seza et al. [35] evaluated a multimodal ResNet-based approach integrating EUS, CT, and MRI/MRP for differentiating serous from mucinous cystic neoplasms, while Nguon et al. [36] used ResNet-50 transfer learning on EUS images for the same diagnostic distinction. Although these studies reported encouraging accuracy, their interpretation remains limited by small or selected cohorts, image augmentation, manual ROI selection, and lack of prospective real-time validation.

AI has also been investigated in the more clinically specific setting of IPMN assessment. Li et al. [37] developed an interpretable EUS feature–based ML model to distinguish main-duct IPMN from chronic pancreatitis in patients with indeterminate main pancreatic duct dilation, with SHAP analysis identifying ductal stones, diffuse lobularity, duct dilation, and atrophy as important predictors. This approach is clinically relevant because it uses structured EUS features rather than raw image analysis, although external and prospective validation are still needed.

Three studies specifically explored AI-assisted EUS approaches for preoperative IPMN risk stratification. Schulz et al. [38] applied transfer learning to EUS images to distinguish low-grade IPMN from high-grade dysplasia or invasive carcinoma, reporting high accuracy in a small dataset that included retrospective, prospective, and external test cases. Krishna et al. [39] and Machicado et al. [40] extended this concept to EUS-guided needle-based confocal laser endomicroscopy, a technique that allows in vivo microscopic assessment during EUS. In these studies, AI-based nCLE analysis improved discrimination of high-risk IPMN features compared with guideline-based or expert interpretation alone. However, the evidence remains preliminary because these studies were based on small, surgically selected, or post hoc cohorts, with limited external validation and no established real-time clinical implementation.

### 3.5. AI-Assisted Anatomical Recognition in Pancreatobiliary EUS and Segmentation

Automatic anatomical recognition and segmentation represent crucial steps toward more reproducible AI-assisted EUS analysis, as manual ROI delineation is time-consuming, operator-dependent, and prone to interobserver variability. By enabling standardized lesion boundary identification, segmentation models may reduce subjective input, improve feature extraction, and facilitate the development of more robust diagnostic and radiomic workflows. Studies on this topic published in the last 5 years are summarized in Supplementary Table S5 [41,42,43,44,45,46,47,48,49].

Several studies have focused on lesion localization and segmentation as preliminary steps for computer-aided detection or downstream quantitative analysis. Konikoff et al. [41] evaluated a DL-based CADe framework for detecting and segmenting pancreatic abnormalities, highlighting the practical relevance of lesion delineation beyond binary classification. Zhang et al. [42] extended this concept to biliopancreatic station recognition and anatomical segmentation, showing that AI may support standardized EUS station classification and pancreas or duct segmentation, although the study remained based on static images rather than live EUS procedures.

Other models specifically addressed pancreatic lesion segmentation across different EUS settings. Li et al. [43] developed a semi-supervised multi-task network for joint lesion segmentation and classification, while Seo et al. [44] applied semantic segmentation to pancreatic cancer and Oh et al. [45] evaluated U-Net-based architectures for pancreatic cystic lesions. The external performance decline observed in the study by Oh et al. [45] is clinically relevant, as it suggests that segmentation models may be sensitive to differences in image acquisition, device characteristics, annotation style, and lesion appearance across datasets.

Iwasa et al. [46] further extended segmentation to CE-EUS video frames, supporting the feasibility of automated tumor delineation in contrast-enhanced imaging, although lesion classification and prospective deployment were not assessed.

More clinically advanced studies have begun to evaluate real-time or procedural applications. Bang et al. [47] prospectively validated PANCRAIEUS, a real-time AI-enhanced EUS system for detecting and segmenting solid pancreatic masses during routine clinical practice, showing high detection performance but still requiring external multicenter and multi-platform validations. Tang et al. [48,49] developed CE-EUS MASTER for real-time capture and segmentation of solid pancreatic masses and explored its use for FNA targeting and trainee education. These studies suggest that segmentation and anatomical recognition may have clinically relevant roles not only in diagnosis but also in procedural standardization, targeting, and training.

## 4. AI Applications in Pancreatic Cytology and EUS-Guided Tissue Acquisition

### 4.1. Cytopathology as Part of the Pancreatic EUS Workflow

Within the pancreatic EUS workflow, EUS-guided FNA and FNB provide the pre-treatment material on which diagnosis, ancillary testing, and increasingly biomarker assessment depend. For solid pancreatic lesions, recent “American Society for Gastrointestinal Endoscopy” and “European Society of Gastrointestinal Endoscopy” guidelines favor contemporary FNB needles over FNA because they more often procure core tissue with preserved architecture, although FNA still has a role when ROSE is available [1,2]. In practice, direct smears or liquid-based cytology are best suited to the evaluation of individual cells and small clusters, whereas cell blocks and biopsy cores provide formalin-fixed paraffin-embedded material for architecture-based histology, immunohistochemistry, and molecular assays. Accordingly, the WHO reporting system advocates close correlation with imaging and encourages the incorporation of ancillary testing into the final cytopathologic diagnosis, including biochemical and molecular testing when appropriate [50]. Cell-block preparation is therefore particularly valuable when differential diagnosis requires lineage-specific markers—such as in PanNETs, solid-pseudopapillary neoplasms, acinar neoplasms, lymphoma, or metastases—and markers such as p53 or SMAD4 may support PDAC in the appropriate morphologic context [51]. Beyond diagnosis, EUS-derived samples, including cell blocks, can support next-generation sequencing or comprehensive genomic profiling when tumor cellularity and tissue area are adequate. These analyses may uncover clinically relevant or drug-matched alterations that can inform treatment planning in selected patients, although the optimal acquisition strategy and real-world success rates remain variable across studies and centers [52,53,54]. Indeed, AI studies in this domain have focused on the same downstream outputs—digitized pancreatic cytology and histology slides, including EUS-FNA-derived cytological images and EUS-FNB-derived histopathological WSI—for assistive triage, cancer detection, and workflow support [55,56,57].

In the present review, evidence on AI-assisted cytopathology is therefore interpreted according to its proximity to the pancreatic EUS workflow. Direct evidence refers to studies in which AI was applied to cytological or histological material obtained through pancreatic EUS-FNA or EUS-FNB. Broader studies on digital cytology, WSI, or pancreatic pathology are used only to provide methodological context and are not presented as direct evidence of AI performance in pancreatic EUS.

Among studies directly connected to pancreatic EUS-guided tissue acquisition, most AI applications have focused on digitized cytological material obtained from EUS-FNA, aiming to support ROSE, improve cancer-cell detection, and reduce dependence on immediate cytopathologist availability. However, this evidence remains less mature than the image-based EUS literature, as most studies are retrospective, based on selected digitized slides or cell clusters, and have limited prospective or real-time validation. Studies on this topic published in the last 5 years are summarized in Supplementary Table S6 [56,57,58,59,60,61,62,63,64,65,66].

From a workflow perspective, AI-assisted ROSE and FNB adequacy assessment may be particularly relevant in centers where immediate cytopathology support is unavailable or inconsistently available. In this setting, AI tools could potentially provide rapid preliminary triage of digitized cytology slides, cell clusters, or specimen images, helping the endoscopist identify inadequate or paucicellular samples, prioritize fields suspicious for malignancy, and decide whether additional needle passes are needed before the procedure is completed. For EUS-FNB, AI-based adequacy assessment could also support specimen triage for cell block preparation, histology, immunohistochemistry, or molecular testing. However, these applications should currently be regarded as workflow-support tools rather than replacements for cytopathologist or pathologist interpretation, as their impact on diagnostic yield, number of passes, repeat procedures, turnaround time, and downstream treatment decisions remains insufficiently validated.

### 4.2. AI-Based Cytology Tools Intended to Support ROSE: From Digital Cytology to Spectral Imaging

In this section, the term “ROSE support” is used cautiously. Most studies retrospectively analyzed previously acquired and digitized cytology images, cell clusters, or tiles; therefore, they should be interpreted as AI-based cytology tools intended to support ROSE rather than as validated real-time ROSE systems. Only prospective evaluation during live EUS-guided sampling can establish whether these tools effectively influence adequacy assessment, provisional diagnosis, and decisions regarding additional needle passes during the procedure.

The largest direct studies in this field evaluated AI-assisted analysis of EUS-FNA cytological images for pancreatic cancer detection. Zhang et al. [57] developed a multicenter DCNN-based segmentation system for stained cell clusters and PDAC identification, showing high image-level and patient-level performance, with results comparable to cytopathologists. Fang et al. [58] subsequently developed a semi-supervised CNN system for PDAC detection on EUS-FNA cytological images, reducing the need for extensive manual annotation and showing encouraging performance across internal and external testing. However, both studies remained retrospective and focused mainly on selected cytology images, while prospective validation within a live ROSE workflow is still lacking.

Smaller proof-of-concept studies have explored more pragmatic cytology workflows. Lin et al. [59] applied a ResNet101V2-based model to digitized Diff-Quik-stained EUS-FNA slides, whereas Tian et al. [60] used mobile phone-captured cytology images and a two-stage DL approach combining tissue-cell detection with malignancy classification. These studies suggest that AI-based cytology support may be feasible even with simplified image-acquisition strategies, but their interpretation remains limited by small cohorts, retrospective design, and lack of real-time procedural validation.

Other studies have focused on technical refinement and speed of cytologic interpretation. Fujii et al. [61] showed that data augmentation, particularly geometric transformation, may improve the performance of transformer-based models in small EUS-FNA cytology datasets. Ashida et al. [62] evaluated rapid AI-based cytologic classification of pancreatic cell clusters and reported short evaluation times, supporting the feasibility of rapid post-acquisition inference. However, this should not yet be considered proof of validated live AI-ROSE, because prospective multicenter evaluation during ongoing EUS-FNA procedures is still needed.

Beyond conventional bright-field cytology, Qin et al. [63,64] combined AI with hyperspectral imaging of EUS-FNA liquid-based cytology specimens. Their studies suggest that HSI-based models may outperform standard RGB-based analysis for PDAC diagnosis and for differentiating PDAC from PanNETs. This approach is conceptually attractive because it extracts spectral information not available from conventional cytology images, but current evidence remains preliminary and based on small retrospective cohorts.

### 4.3. AI in EUS-FNB Specimen Adequacy and Histopathological Assessment

While the studies discussed above mainly address cytological interpretation, AI has also been applied to later steps of the EUS-guided tissue workflow, including assessment of FNB specimen adequacy and digital histopathological analysis.

Ishikawa et al. [65] evaluated AI for assessing the adequacy of EUS-FNB specimens in pancreatic diseases using stereomicroscopic images of fresh samples. An initial AlexNet model tested on 98 specimens achieved lower accuracy than expert macroscopic on-site evaluation (MOSE), whereas contrastive learning linking stereomicroscopic and hematoxylin and eosin-stained images improved performance to a level comparable with expert MOSE. These findings suggest that AI may support more objective specimen triage, although the study remained retrospective, single-center, and lacked external or prospective validation.

After specimen acquisition and adequacy assessment, AI has also been applied to the histopathological interpretation of samples. Naito et al. [56] developed an EfficientNet-B1 CNN to detect PDAC on histopathological WSI from EUS-FNB specimens, reporting high diagnostic performance. Although promising, these results should be interpreted cautiously because the test set was restricted to cases with complete diagnostic agreement among three pancreatic pathologists, potentially enriching the evaluation set for diagnostically clearer cases; moreover, false negatives occurred mainly with very small cancer foci, and external validation was lacking.

Beyond slide-level PDAC detection, Udriștoiu et al. [66] evaluated U-Net-based segmentation architectures for automatic localization of malignant tissue on EUS-FNB WSI. Cross-dataset testing showed lower but still promising performance, supporting the feasibility of tumor segmentation in digital histology while also emphasizing the need for standardized annotation, larger multicenter datasets, and prospective workflow-level evaluation.

### 4.4. Critical Appraisal Across Study Domains

Overall, the available evidence suggests that AI applications in pancreatic EUS are technically feasible across multiple diagnostic steps, but the strength of evidence varies substantially according to clinical task, model type, dataset structure, and validation strategy. CNN- and DL-based models predominate in lesion detection, differential diagnosis, segmentation, ROSE, and digital pathology, whereas radiomics, ultrasomics, and conventional ML approaches have been more frequently applied to PanNET characterization, functional subtyping, and grading prediction. However, direct comparison of diagnostic performance across studies remains difficult because the models address heterogeneous tasks, including lesion detection, binary or multiclass classification, anatomical recognition, procedural quality control, cytology triage, specimen adequacy assessment, and WSI-based tumor detection.

A major methodological issue is that dataset size is often reported in terms of images, frames, cytological fields, or histological patches rather than independent patients. Large image numbers may therefore originate from relatively small patient cohorts and may not fully capture patient-level variability. In addition, image-level, frame-level, or patch-level splitting without clear patient-level separation may introduce data leakage, particularly when visually similar frames from the same EUS examination or multiple patches from the same slide are distributed across training and testing sets. Similar leakage may occur when data augmentation or synthetic oversampling is performed before dataset partitioning, because augmented versions of the same original image, frame, cell cluster, tile, or patch may be assigned to both training and test sets. Therefore, patient-level splitting should be performed before any augmentation, oversampling, feature selection, or model-training procedure.

Other recurrent methodological practices, including extensive image augmentation, synthetic oversampling techniques such as SMOTE, and manual ROI segmentation, may further inflate reported performance if not applied within a strictly separated training framework and validated externally. This concern is especially relevant when these approaches are combined with small single-center cohorts, class imbalance, selected still images, and lack of independent validation. These recurring limitations partly reflect the intrinsic complexity of pancreatic EUS research, including operator- and platform-dependent image acquisition, lesion heterogeneity, limited representation of rare but clinically relevant subgroups, variable reference standards, and the need for labor-intensive expert annotation of EUS, cytology, and pathology datasets. Consequently, very high accuracy or AUC values from retrospective studies should be interpreted cautiously and regarded as proof-of-concept rather than evidence of clinical readiness, especially when derived from small cohorts, selected images, restricted disease spectra, or non-independent image-, frame-, tile-, or patch-level analyses. In this context, test-cohort, external, cross-dataset, and subgroup performance are more informative than peak training metrics. This is illustrated by several PanNET radiomics and ultrasomics studies showing lower test than training performance, by segmentation models with reduced external or cross-dataset performance, and by lower performance in clinically difficult settings such as atypical cytology, small lesions, unclear lesion boundaries, sparsely represented high-grade tumors, or very small malignant foci. Similarly, histopathology models tested only on diagnostically concordant cases should be interpreted cautiously, because exclusion of discordant cases may enrich the test set for easier examples. Studies with patient-level validation, external testing, prospective or real-time evaluation, and measurable improvement in physician performance provide stronger evidence for clinical translation than standalone retrospective classifiers.

To facilitate interpretation of this heterogeneous evidence, a qualitative narrative summary of the main AI applications in pancreatic EUS according to clinical task, validation status, and clinical-readiness interpretation is provided in Table 1.

## 5. Future Directions: Toward an Integrated AI-Assisted Pancreatic EUS Workflow

The emergence of prospective reader studies evaluating human–AI collaboration marks an important transition from proof-of-concept algorithm development toward clinically deployable AI systems capable of supporting endosonographers throughout the entire EUS workflow. While early research primarily focused on developing algorithms capable of detecting and characterizing pancreatic lesions, the field is progressively shifting toward clinically deployable systems designed to support physicians throughout the entire EUS examination. The next generation of AI systems will likely function as comprehensive procedural assistants rather than isolated diagnostic tools. Achieving this vision will require not only further improvements in algorithm performance but also seamless clinical integration, prospective validation, and demonstration of meaningful benefits for both physicians and patients.

### 5.1. Multimodal AI Models Combining Imaging, Cytology, Pathology, and Clinical Data

Most currently available models rely on a single data source, such as static EUS images, selected video frames, cytology slides, stereomicroscopic images of fresh FNB samples, or histopathological WSI [23,24,25,26,56,61,65,66]. Future systems should instead combine complementary information from B-mode EUS, EUS video, CE-EUS, elastography, cytology, histology, clinical variables, biomarkers, CT/MRI, and longitudinal outcome data. This would better reflect real clinical reasoning, in which lesion morphology, vascularity, stiffness, tissue adequacy, cytological findings, histological architecture, and patient-level risk factors are interpreted together rather than in isolation.

The studies by Mo et al. already suggest that combining quantitative EUS-derived features with clinical or semantic EUS variables can improve model performance compared with imaging signatures alone [23,24,25,26]. Similarly, AI-assisted cytology and ROSE models show the potential to support rapid cellular interpretation, while models applied to FNB specimens and digital pathology may provide downstream confirmation of adequacy, malignancy, or tumor segmentation [56,61,65,66]. A future multimodal architecture could therefore link upstream EUS image analysis with downstream cytological and histological ground truth, creating a feedback loop in which tissue-based data improve image-based prediction and procedural targeting.

Such integration, however, requires more than model development. It will require standardized image acquisition, harmonized annotation across frames, ROIs, cytology images, tissue cores, and WSI, as well as robust data linkage at the patient level. Particular attention should be given to vendor-related variability, staining and preparation differences, missing clinical data, and temporal alignment between imaging, pathology, treatment, and outcomes. Explainability tools such as Grad-CAM and SHAP may help clinicians understand model behavior, but they should complement, rather than replace, rigorous external validation, calibration assessment, and clinical impact studies [26].

### 5.2. Real-Time Decision Support During EUS Procedures

A major future direction is the transition from retrospective image classification to real-time decision support during EUS procedures. In this setting, AI could assist the endosonographer by identifying the most suspicious or viable area of a lesion, supporting needle targeting, estimating the probability of malignancy or high-grade disease, suggesting whether additional sampling is needed, and providing immediate adequacy feedback. This would be particularly relevant in centers where on-site cytopathology is unavailable or inconsistently accessible.

Current evidence provides early proof-of-concept for several components of this workflow. AI-assisted ROSE models have been developed to detect malignant cytological clusters and support rapid cytology interpretation [57], while AI applied to stereomicroscopic images of fresh EUS-FNB samples has shown potential for objective specimen adequacy assessment [65]. Downstream, digital pathology models have demonstrated the feasibility of detecting or segmenting PDAC on EUS-FNB WSI [66]. Taken together, these applications suggest that AI could eventually support a continuous procedural pathway: lesion assessment, targeting, sampling, adequacy confirmation, cytological interpretation, and histopathological analysis.

For this to become clinically meaningful, future AI tools must be designed around the realities of the EUS suite. Models should provide results with minimal latency, integrate into existing endoscopy reporting systems, avoid interrupting the procedure, and present outputs as calibrated probabilities with clear uncertainty estimates. Visual explanations should be simple enough to be interpreted during the procedure, and the operator should retain the ability to override AI suggestions. Importantly, real-time AI should remain assistive rather than autonomous, with clinical responsibility clearly maintained by the endosonographer and pathology team.

### 5.3. Methodological, Ethical, and Implementation Challenges for AI in Pancreatic EUS

At the review level, some methodological limitations should also be acknowledged. This article was designed as a clinically oriented narrative review rather than a systematic review or meta-analysis; therefore, no formal PRISMA-based screening process, study-level risk-of-bias assessment, or quantitative quality grading was performed. In addition, the literature search was restricted to PubMed and to articles published within the previous five years. Although this approach was chosen to provide an up-to-date synthesis focused on clinically relevant pancreatic EUS applications, it may have introduced selection bias and may have excluded earlier proof-of-concept studies or technical AI and image-processing studies indexed only in engineering-focused or broader biomedical databases, such as IEEE Xplore, Scopus, Web of Science, or Embase.

Although AI may enhance lesion characterization, tissue acquisition, cytology interpretation, and histopathological support in pancreatic EUS, its translation into routine practice remains limited by methodological, ethical, regulatory, and infrastructural barriers. These limitations are particularly relevant in pancreatic EUS, where most available studies are retrospective, frequently single-center, and based on relatively small datasets, manually selected images, manual ROI segmentation, or selected cytology and histology samples [23,24,25,26,56,61,65,66].

From a methodological perspective, data leakage, overfitting, and optimism remain major concerns. This is particularly relevant when multiple images, frames, or patches are extracted from the same patient, because image-level or frame-level splitting without clear patient-level separation may lead to data leakage and overoptimistic estimates of model performance. For radiomics and ultrasomics models, these concerns are amplified by the high-dimensional nature of extracted features and by the sensitivity of texture and higher-order descriptors to acquisition parameters, preprocessing, ROI segmentation, feature-selection strategy, and harmonization procedures. Many radiomics, ultrasomics, and DL studies extract high-dimensional features from limited institutional cohorts, with variable reporting of data splitting, feature selection, hyperparameter tuning, and testing procedures [23,24,25,26]. Cross-validation can reduce internal instability, but it cannot replace independent validation across different EUS platforms, processors, operators, acquisition settings, staining protocols, cytology workflows, and pathology pipelines [19,27]. This is especially important because AI models trained on selected still images may not reproduce the complexity of real-time EUS, where image quality, lesion visualization, artifacts, and operator-dependent scanning patterns vary substantially.

Class imbalance, incomplete data, and heterogeneous reference standards further affect reliability. Pancreatic EUS studies often include uneven distributions of disease categories, tumor grades, cytological diagnoses, or histological outcomes. In addition, key variables such as Ki-67, molecular markers, treatment history, cytology adequacy, number of needle passes, histological confirmation, and follow-up outcomes may be unavailable or inconsistently reported. Reference labels may also vary across studies, ranging from surgical pathology to EUS-FNA/FNB, cytology, follow-up, or expert consensus. These inconsistencies can introduce noise into model training and make comparisons across studies difficult.

Current AI tools also remain insufficient for some clinically decisive tasks. Image-based models may assist differential diagnosis or PanNET grading prediction, but they cannot yet replace tissue acquisition when cytology, histology, immunohistochemistry, Ki-67 assessment, or molecular profiling is required for management [27,28,29,30]. Similarly, AI-assisted ROSE, specimen adequacy assessment, and WSI-based histopathology models are promising, but they remain adjunctive tools rather than substitutes for cytopathologist or pathologist interpretation [56,61,65,66]. At present, AI in pancreatic EUS should therefore be framed as decision support across the diagnostic workflow, not as an autonomous diagnostic authority.

Future evaluation should also move beyond discrimination metrics alone. AUC and accuracy are useful, but they do not capture calibration, uncertainty, subgroup performance, failure modes, or dataset shift. For pancreatic EUS, clinically meaningful endpoints should include whether AI reduces non-diagnostic sampling, decreases the number of needle passes, improves lesion targeting, shortens time to diagnosis, increases agreement between endosonographers and pathologists, or changes therapeutic decision-making. Pragmatic prospective studies, ideally followed by randomized or stepped-wedge trials, will be needed to determine whether AI-assisted EUS provides measurable clinical benefit rather than only retrospective diagnostic performance.

Ethical and governance issues are equally relevant. Developing robust AI models requires large, well-annotated, and diverse datasets, including EUS images or videos, cytology images, FNB specimen images, WSI, clinical variables, and follow-up outcomes. Such integration raises questions about consent, data ownership, secondary use of clinical images, data sharing, and patient privacy [67,68]. The risk of re-identification may increase when EUS data are linked with pathology, molecular information, and longitudinal outcomes. Privacy-preserving strategies, including federated learning, and transparent multicenter data governance may help support collaboration while limiting unnecessary transfer of sensitive patient-level data [69].

Algorithmic bias is another barrier to clinical adoption. If training datasets are derived mainly from expert centers, selected devices, specific staining protocols, or highly experienced operators, performance may decline in lower-volume centers or different healthcare settings [70,71]. Bias may also arise from under-representation of specific lesion types, small tumors, atypical cytology, inflammatory mimics, cystic components, or technically difficult sampling conditions. For this reason, future studies should report subgroup performance, include heterogeneous multicenter datasets, and evaluate model behavior across devices, operators, lesion characteristics, and specimen-preparation workflows.

Interpretability and professional accountability will also shape implementation. Explainability tools such as Grad-CAM, SHAP, LIME, or saliency-based methods may help clinicians understand which image regions or features influence model predictions, but these outputs should be interpreted cautiously and validated against clinical and pathological reasoning. In the EUS suite, there is also a risk of automation bias, particularly if AI outputs are presented as definitive rather than probabilistic [71]. User interfaces should therefore provide calibrated probabilities, uncertainty estimates, clear visual feedback, and the possibility for the endosonographer, cytopathologist, or pathologist to override AI suggestions.

Finally, clinical deployment will require regulatory clarity, interoperability, and sustainable infrastructure. Integrated AI-assisted pancreatic EUS would depend on reliable storage and linkage of EUS videos, DICOM images, cytology images, FNB specimen images, WSI, and clinical data, together with version control, audit trails, cybersecurity, privacy protection, and post-deployment monitoring. These requirements entail costs for secure data storage, software maintenance, workflow integration, and staff training, which may disadvantage smaller or resource-limited centers if adoption is not planned equitably [72]. Regulatory frameworks such as the U.S. “Food and Drug Administration” guidance on Predetermined Change Control Plans, the “European Union’s Artificial Intelligence Act”, and the “European Health Data Space Regulations” emphasize predefined update pathways, transparency, risk management, post-market surveillance, human oversight, interoperability, and responsible secondary use of health data [73,74,75]. Overall, AI tools in pancreatic EUS should not be evaluated only by retrospective accuracy metrics but also by their safety, governance, feasibility, and capacity to function as transparent, validated, and workflow-compatible decision-support systems.

## 6. Conclusions

AI is an emerging area of research in pancreatic EUS, with potential applications ranging from image-based lesion detection and differential diagnosis to segmentation, procedural quality control, ROSE, cytology, specimen adequacy assessment, and digital histopathology. Current studies suggest technical feasibility and promising diagnostic performance in selected settings, but the overall evidence remains preliminary.

Most available studies are retrospective and heterogeneous, frequently based on selected still images, manually segmented ROIs, limited sample sizes, single-center datasets, image-level analyses, and insufficient external or prospective validation. Therefore, current AI models should not be considered clinically established tools or substitutes for expert endosonographic assessment, EUS-guided tissue acquisition, cytopathology, or histopathology. Their most appropriate role at present is as investigational decision-support systems requiring further validation.

Integrated and personalized AI-assisted pancreatic EUS workflows should therefore be regarded as a future possibility rather than an established clinical benefit. Clinical translation will require standardized acquisition and annotation protocols, strict patient-level validation, independent multicenter testing, prospective or real-time evaluation, explainable and calibrated outputs, robust data governance, and demonstration of measurable impact on clinically relevant endpoints such as diagnostic yield, sampling adequacy, time to diagnosis, number of needle passes, and treatment decision-making.

## Figures and Tables

**Table 1:** Narrative summary of AI applications in pancreatic EUS by clinical task, validation status, and clinical-readiness interpretation.

Clinical Task	Predominant Evidence Base	Validation Status	Clinical-Readiness Interpretation
Lesion detection and quality control	DL/CNN systems for anatomical station recognition, lesion detection, documentation completeness, and procedural quality support	Retrospective validation, with external, prospective, or real-time evaluation available in selected studies	Among the more mature applications; potentially useful for workflow support and quality control, but broader multicenter implementation studies are still needed
Differential diagnosis of solid pancreatic lesions	CNN/DL and multimodal models for PDAC, AIP, PanNETs, and other solid pancreatic lesions	Mostly retrospective image-based studies, with some external validation, reader-assistance, and video-based evaluation	Promising as decision support, but not ready for autonomous diagnosis
PanNET characterization and grading	Radiomics, ultrasomics, DL signatures, and combined nomograms	Mainly retrospective, often small or single-center cohorts; frequent manual ROI segmentation; limited external validation	Preliminary and hypothesis-generating; not sufficient to replace histology, Ki-67 assessment, or multidisciplinary evaluation
Pancreatic cystic lesions and IPMN risk stratification	CNN/DL models based on EUS images, multimodal imaging, and EUS-nCLE	Mostly small retrospective or post hoc datasets; limited prospective validation	Preliminary adjunct to guideline-based assessment; clinical integration remains unproven
Anatomical recognition, segmentation, and procedural guidance	CADe, U-Net-based segmentation, station recognition, and real-time capture systems	Heterogeneous evidence, including selected prospective or real-time studies	Emerging for standardization, training, and targeting support; impact on diagnostic outcomes remains to be demonstrated
AI-assisted ROSE, cytology, specimen adequacy, and histopathology	DL models applied to EUS-FNA/FNB-derived cytology, cell clusters, specimen images, or WSI	Mostly retrospective and proof-of-concept, with limited prospective real-time validation	Early-stage; potentially useful for triage and workflow support, but still adjunctive to cytopathologist/pathologist interpretation
Integrated multimodal EUS-to-pathology workflow	Proposed integration of EUS imaging, tissue acquisition, cytology/histology, and clinical data	No fully validated integrated system identified	Future direction rather than current clinical reality

Note: Clinical-readiness interpretation represents a qualitative narrative appraisal based on the type of clinical task, validation strategy, proximity to the real-time EUS workflow, and availability of external or prospective evaluation. It should not be interpreted as a formal grading system. AI, artificial intelligence; EUS, endoscopic ultrasonography; DL, deep learning; CNN, convolutional neural network; PDAC, pancreatic ductal adenocarcinoma; AIP, autoimmune pancreatitis; PanNET, pancreatic neuroendocrine tumor; IPMN, intraductal papillary mucinous neoplasm; nCLE, needle-based confocal laser endomicroscopy; CADe, computer-aided detection; ROSE, rapid on-site evaluation; FNA, fine-needle aspiration; FNB, fine-needle biopsy; WSI, whole-slide imaging.

## Data Availability

No new data were created or analyzed in this study. Data sharing is not applicable to this article.

## Supplementary Materials

The following supporting information can be downloaded at: https://www.mdpi.com/article/10.3390/jcm15187167/s1, Table S1: Inclusion and Exclusion Criteria for the Narrative Literature Review; Table S2: AI-assisted image-based lesion detection, classification, and quality control in pancreatic EUS; Table S3: AI-assisted EUS for differential diagnosis, characterization, and grading of solid pancreatic lesions; Table S4: AI-assisted EUS and EUS-nCLE for differential diagnosis and risk stratification of pancreatic cystic lesions; Table S5: AI-assisted anatomical recognition, lesion segmentation, and procedural guidance in pancreaticobiliary EUS; Table S6: AI applications in cytology, specimen adequacy assessment, and histopathology using pancreatic EUS-FNA/FNB-derived material.

## Abbreviations

The following abbreviations are used in this manuscript:

EUS	Endoscopic ultrasound
FNA	Fine needle aspiration
FNB	Fine needle biopsy
PDAC	Pancreatic ductal adenocarcinoma
PanNET	Pancreatic neuroendocrine tumor
AIP	Autoimmune pancreatitis
ROSE	Rapid on-site evaluation
AI	Artificial intelligence
DL	Deep learning
CNN	Convolutional neural network
WSI	Whole-slide imaging
CAD	Computer-assisted diagnosis
DCNN	Deep convolutional neural network
DSC	Dice similarity coefficient
ResNet	Residual network
PCL	Pancreatic cystic lesions
AUC	Area under the curve
CE-EUS	Contrast-enhanced EUS
LSTM	Long short-term memory
ML	Machine learning
SVM	Support vector machine
PPV	Positive predictive value
NPV	Negative predictive value
ROI	Region of interest
RGB	Red, green, blue
SHAP	SHapley Additive exPlanations
RF	Random forest
LASSO	Least absolute shrinkage and selection operator
SMOTE	Synthetic minority over-sampling Technique
WHO	World Health Organization
XGBoost	Extreme gradient boosting
CT	Computed tomography
MRI	Magnetic resonance imaging
IPMN	Intraductal papillary mucinous neoplasms
nCLE	Confocal laser endomicroscopy
CADe	Computer-aided detection
SSM-Net	Semi-supervised multi-task network
IoU	Intersection over union
HSI	Hyperspectral imaging
AGF	Attribution guided factorization
H&E	Hematoxylin and eosin
MOSE	Macroscopic on-site evaluation
