# Supplementary material for: Artificial Intelligence in Pancreatic Endoscopic Ultrasonography: From Image-Based Diagnosis to Cytopathology
Source: J Clin Med. 2026 Sep 15;15(18):7167. doi: 10.3390/jcm15187167 (PMC13607084; doi:10.3390/jcm15187167)
Supplement: Supplementary file 1 [file jcm-15-07167-s001.zip › jcm-4477041-supplementary/jcm-4477041-supplementary.pdf]

**Table S1.** Inclusion and Exclusion Criteria for the Narrative Literature Review

| Eligibility Domain | Inclusion Criteria | Exclusion Criteria |
|---|---|---|
| Article type | Original research articles reporting primary data | Reviews, meta-analyses, editorials, letters, correspondence, commentaries, opinion pieces, and guidelines |
| Clinical focus | Studies focused on pancreatic diseases evaluated by EUS or EUS-guided tissue acquisition | Studies not focused on pancreatic diseases |
| AI application | Studies investigating AI, machine learning, or deep learning as a core analytical component | Studies in which AI was not directly applied or was only discussed conceptually |
| Diagnostic domain | Studies addressing EUS image-based diagnosis or EUS-guided cytological/ histological assessment | Studies unrelated to EUS imaging, EUS-FNA/FNB, cytology, or pathology |
| Publication period | Articles published between 2021 and 2026 | Articles published outside the predefined period |
| Publication format | Full-text peer-reviewed articles | Conference abstracts, meeting proceedings, and non-peer-reviewed material |

EUS, endoscopic ultrasonography; AI, artificial intelligence; FNA, fine needle aspiration; FNB, fine needle biopsy

**Table S2.** AI-assisted image-based lesion detection, classification, and quality control in pancreatic EUS

| Study | Design and validation | Dataset | AI Method | Diagnostic Task | Main Findings | Limitations |
|---|---|---|---|---|---|---|
| Li 2025 [8] | Retrospective internal and external testing plus single-center prospective observational testing; validation performed for an EUS-AIRS quality-control/reporting system; patient-level split for model development not reported | - Patients/cases: internal test, 107 EUS procedures; external test, 50 EUS procedures; prospective test, 114 patients<br>- Data units: 235,784 EUS images used to train/test eight DCNN models | EUS-AIRS: real-time automatic image reporting system integrating eight DCNNs | Automated EUS photodocumentation and quality control: standard stations, lesions, puncture procedures, and lesion segmentation | - Completeness of standard-station documentation: 90.8% vs. 70.5% in internal testing, 91.4% vs. 68.2% in external testing, and 91.4% vs. 78.1% in prospective testing for EUS-AIRS vs. endoscopists, respectively<br>- Standard-station capture accuracy: 90.3% internal, 89.3% external, and 88.2% prospective<br>- Lesion segmentation DSC: 0.86 internal, 0.83 external, and 0.84 prospective | - Prospective test single-center<br>- Retrospective external testing available but no multicenter prospective clinical validation<br>- Limited lesion spectrum<br>- Focused on photodocumentation/quality control rather than diagnostic classification |
| Saraiva 2024 [12] | Retrospective multicenter transatlantic study; 90:10 training/testing split; patient-level split not reported; no prospective real-time validation | - Patients/cases: 378 EUS examinations from four referral centers<br>- Data units: 126,000 EUS images | CNN based on ResNet; 90:10 training/testing split | Differentiation of cystic and solid pancreatic lesions on EUS | Accuracy: 99.0% for mucinous PCL; 99.8% for non-mucinous PCL; 94.0% for PDAC vs. PanNET | - Retrospective design<br>- Still-image analysis<br>- Patient-level split not reported<br>- Possible overfitting<br>- Prospective real-time validation needed |
| Zhang 2025 [11] | Retrospective single-center study; internal model development/testing; split level not reported; no external or prospective validation reported | - Patients/cases: 425 patients<br>- Data units: B-mode EUS images; elastography available only in a subset; exact image/frame count not reported | Multimodal DCNN system using B-mode EUS, EUS elastography, and clinical data | Lesion detection and segmentation; carcinoma vs. non-cancerous lesions | B-mode + EUS elastography: accuracy 94.0%, AUC 0.94 | - Single-center, retrospective design<br>- Elastography available only in a subset<br>- Limited multimodal test set<br>- Need for prospective multicenter validation |
| Podină 2026 [10] | Prospective multicenter observer-blinded video-based study; standardized anonymized CE-EUS | Patients/cases: 118 histologically confirmed solid pancreatic masses | CNN-based segmentation + time–intensity curve extraction + | PDAC vs. non-PDAC | - Dedicated AI system: accuracy 95.8%, sensitivity 96.6%, specificity 94.1%<br>- Human readers: accuracy 67.2%-80.8% | - Single platform/contrast agent<br>- Selected high-quality videos<br>- Limited external generalizability |

| | | | | | | |
|---|---|---|---|---|---|---|
| | videos assessed by human readers and compared with a dedicated AI system; ChatGPT included only as an exploratory general-purpose comparator; no live real-time AI-assisted decision-making | - Data units: 118 standardized 2-min CE-EUS videos | feedforward neural network classification; ChatGPT comparator | | - ChatGPT comparator: correct identification of PDAC in 14.1% of cases | - No evaluation of live real-time AI-assisted decision-making |
| Udriștoiu 2021 [13] | Retrospective single-center pilot image-based study; internal image-level training/testing after image selection and augmentation; patient-level separation not reported; no independent external validation | - Patients/cases: 65 patients<br>- Data units: 20 images selected per patient across grayscale EUS, color Doppler, CE-EUS, and elastography; 2688 training images and 672 testing images after augmentation | Hybrid CNN-LSTM model integrating gray-scale EUS, color Doppler, CE-EUS, and real-time elastography | PDAC vs. chronic pseudotumoral pancreatitis vs. PanNET | - Accuracy: 98.3%<br>- Sensitivity: 98.6%<br>- Specificity: 97.4%<br>- AUC: 0.98 | - Small single-center pilot cohort<br>- Image-level analysis<br>- Patient-level separation not reported<br>- No independent external validation<br>- Proof-of-concept design |
| Wu 2023 [9] | Prospective single-center randomized controlled trial of real-time EUS-IREAD assistance; model development/testing included internal and external image datasets and 60 EUS videos; no multicenter clinical validation | - Patients/cases: 290 randomized patients: 144 EUS-IREAD-assisted and 146 controls<br>- Data units: training/internal/external EUS image datasets for station recognition and anatomical-structure localization + 60 EUS videos for video testing | EUS-IREAD real-time AI device integrating five DCNN models for eight-station recognition and localization of 24 anatomical structures | - Real-time quality control during linear biliopancreatic EUS<br>- Reduced missed standard stations and anatomical structures | - Missed standard-station scanning rate: 4.5% vs. 14.3% in controls; absolute difference −9.8%<br>- Missed anatomical-structure scanning rate: 6.2% vs. 17.4% in controls<br>- No increase in procedure time | - Single-center clinical trial<br>- Olympus platforms only<br>- Focused on procedural completeness and quality control<br>- Multicenter validation needed |

AI, artificial intelligence; EUS, endoscopic ultrasonography; EUS-AIRS, EUS automatic image report system; DCNN, deep convolutional neural network; DSC, Dice similarity coefficient; CNN, convolutional neural network; ResNet, residual network; PCL, pancreatic cystic lesion; PDAC, pancreatic ductal adenocarcinoma;

PanNET, pancreatic neuroendocrine tumor; AUC, area under the curve; CE-EUS, contrast-enhanced endoscopic ultrasonography; LSTM, long short-term memory; EUS-IREAD, EUS-intelligent and real-time endoscopy analytical device

**Table S3.** AI-assisted EUS for differential diagnosis, characterization, and grading of solid pancreatic lesions

| Study | Design and validation | Dataset | AI Method | Diagnostic Task | Main Findings | Limitations |
|---|---|---|---|---|---|---|
| **Retrospective Studies (PDAC)** | | | | | | |
| Ruano 2022 [14] | Retrospective, single-center study; 10 random splits; full-procedure frame-based analysis; patient-level split not reported; no external validation | - Patients/cases: 55 cancer participants + 37 non-cancer subjects<br>- Data units: 16,585 cancer frames + 49,664 non-cancer frames | Handcrafted multiscale texture features; SVM and AdaBoost; 10 random splits | Pancreatic cancer detection across full EUS procedures | - Accuracy: 92.1%<br>- Sensitivity: 96.3%<br>- Specificity: 87.8% | - Retrospective, single-center design<br>- Small patient cohort<br>- No external validation |
| Kuwahara 2023 [15] | Retrospective, single-center study with temporally separate test cohort; patient-level test performance reported; no prospective real-time or external validation | - Patients/cases: 933 patients<br>- Data units: 22,000 training images + 3171 test images | EfficientNetV2-L DL model; DCGAN image generation | Carcinoma vs. non-carcinoma classification across multiple pancreatic mass types | - Patient-level test performance: AUC: 0.90<br>- Accuracy: 0.91<br>- Sensitivity: 0.94<br>- Specificity: 0.82<br>- PPV: 0.93<br>- NPV: 0.84 | - Retrospective, single-center design<br>- Possible selection bias<br>- No prospective real-time external validation |
| Tian 2022 [16] | Retrospective single-center study; patient-level random allocation into training, validation, and test groups at approximately 4:1:1; EUS images extracted from videos; manual ROI annotation; no external validation | - Patients/cases: 157 patients: 102 pancreatic cancer cases and 55 non-pancreatic cancer cases<br>- Data units: 1213 EUS images extracted from EUS videos, approximately 1–20 images per patient | YOLOv5m object detection model with manual ROI annotation | Real-time detection and classification of pancreatic cancer vs. non-pancreatic cancer lesions | - AUC: 0.85<br>- Sensitivity: 95%<br>- Specificity: 75%<br>- Performance not significantly superior to physicians | - Retrospective, single-center design<br>- Small validation/test samples<br>- No external validation<br>- Only YOLOv5m tested |
| Hu 2024 [17] | Retrospective, multicenter image-based model-development study with external image validation and physician comparison; split level not reported as patient-level; no video or prospective validation | - Patients/cases: 155 patients in Dataset A; patient number for Dataset B not reported<br>- Data units: Dataset A: 1500 cancer + 300 non-cancer EUS images; Dataset B: 1000 external EUS images, including 585 cancer + 415 non-cancer images | Modified Faster R-CNN with CLAHE preprocessing, ResNet-50 feature extraction, feature fusion, ROI Align, and receptive field block | - Detection of pancreatic cancer on EUS images<br>- Comparison with expert and novice physicians | External validation: AUC 0.92, sensitivity 91.7%, specificity 91.5%, accuracy 91.6%, detection time 113 ms/image | - Retrospective design<br>- Still-image design<br>- No video validation<br>- Limited disease spectrum (cancer vs. non-cancer) |
| Marya 2021 [18] | Retrospective, single-center study with expert comparison; image/video-derived dataset; split level not | - Patients/cases: 583 patients<br>- Data units: 1,174,461 EUS images from still images and videos | ResNet50V2 EUS-CNN; occlusion heatmap analysis; comparison | AIP vs. PDAC | - Sensitivity: 90%<br>- Specificity: 93%<br>- AUC: 0.96 | - Retrospective, single-center design<br>- Not tested in real-time workflow |

| | | | | | | |
|---|---|---|---|---|---|---|
| | reported as patient-level; no prospective real-time validation | | with 7 expert endosonographers | | | - No prospective validation |
| Nakamura 2024 [19] | Retrospective single-center study; patient-level 5-fold cross-validation framework using ordered and balanced datasets; quasi-RGB images generated from EUS video frames; no external or prospective validation | - Patients/cases: 85 patients: 24 localized AIP and 61 PDAC<br>- Data units: 8751 AIP EUS images and 20,584 PDAC EUS images | ResNet152 with transfer learning; quasi-RGB images from EUS video frames; data augmentation, ensemble learning, 5-fold cross-validation | Localized AIP vs. PDAC | - AUC: 0.85<br>- Accuracy: 0.80<br>- ResNet152 outperformed expert and novice endoscopists | - Retrospective, single-center design<br>- Small dataset<br>- No prospective external validation |
| **Prospective and Reader-Assistance Studies (PDAC)** | | | | | | |
| Gu 2023 [20] | Retrospective single-center model development with patient-level random training/validation split; prospective test cohort and reader-assistance study; ROI-based still-image analysis; no external validation | - Patients/cases: 491 patients/lesions total: 368 retrospective development/validation patients and 123 prospective test patients<br>- Data units: conventional two-dimensional EUS ROI images, usually 5–10 images per patient; exact total image count not reported | ResNet50-based DL radiomics model using EUS ROI images; AI scores and heatmaps | - PDAC vs. benign pancreatic lesions<br>- AI-assisted interpretation by endosonographers | - Prospective cohort: AUC 0.94, accuracy 0.86, sensitivity 0.83, specificity 0.90<br>- AI ↑ overall reader performance | - Single-center data<br>- Still-image analysis<br>- No video-based validation |
| Cui 2024 [21] | Retrospective multicenter model development with internal testing and three external test datasets; prospective randomized crossover reader trial; image-based model testing and patient-level reader-assistance evaluation; EUS image-based rather than live real-time procedural evaluation | - Patients/cases: 628 retrospective patients: 439 from one institution for training/internal testing and 189 from three external institutions; prospective crossover cohort of 130 patients and 12 endoscopists<br>- Data units: 6181 EUS images for training/validation, 1545 EUS images for internal testing, 1205 external still images, plus clinical variables | Multimodal joint-AI model: ResNet-50 EUS-CNN + ML models using clinical variables; multilayer perceptron fusion; Grad-CAM and SHAP interpretability | - Carcinoma vs non-cancerous solid pancreatic lesions<br>- AI-assisted diagnosis by endoscopists | - Joint-AI AUC: 0.99 in the internal test dataset; 0.95, 0.92, and 0.97 in the three external test datasets<br>- In novices, ↑ accuracy: 0.69 → 0.90; ↑ sensitivity: 0.61 → 0.91 | - EUS images rather than real-time procedures<br>- Prospective cohort limited to 130 patients<br>- Further real-world validation needed |

| | | | | | | |
|---|---|---|---|---|---|---|
| Montanelli 2026 [22] | Randomized controlled tandem video reader study; AI-assisted vs. unassisted dynamic video interpretation; reader-level evaluation; no prospective live clinical validation | - Patients/cases: 16 standardized EUS video clips from complete pancreatic examinations; 32 endosonographers<br>- Data units: 16 EUS video clips | mAI Companion© AI software system | AI-assisted detection and localization of small solid pancreatic tumors during dynamic EUS video interpretation | Stand-alone AI accuracy: 93.8%; reader accuracy increased from 70.5% without AI to 82.0% with AI assistance (p<0.001) | - Video-clip reader study rather than live real-time EUS<br>- Limited number of cases<br>- Structured interpretation setting<br>- No prospective clinical validation |
| **Retrospective Studies (PanNET)** | | | | | | |
| Yi 2025 [3] | Retrospective single-center study; patient-level random training/test split at 7:3; manual ROI segmentation; no external or prospective validation | - Patients/cases: 266 patients: 115 PanNETs and 151 pancreatic cancers<br>- Data units:<br>EUS ROI images/features; exact image count not reported | ResNet18 feature extraction; 2048 DL features, 27 retained; SVM selected; Grad-CAM and SHAP | PanNETs vs. pancreatic cancer | - SVM-based DL model: AUC 0.95 training, 0.79 test<br>- Test accuracy: 0.77<br>- Test sensitivity: 0.80<br>- Test specificity: 0.74<br>- Nomogram: AUC 0.96 training, 0.87 test | - Retrospective, single-center design<br>- Limited sample size<br>- Manual ROI segmentation<br>- Heterogeneous EUS devices<br>- No external/prospective validation |
| Mo 2024 [23] | Retrospective single-center EUS-based ultrasomics study; patient-level random training/test split at 7:3; manual ROI delineation; no external or prospective validation | - Patients/cases: 231 patients: 127 pancreatic cancer and 104 PanNETs<br>- Data units: conventional EUS ROI images; 107 ultrasomics features extracted; exact image count not reported | Conventional EUS ultrasomics; 107 features extracted, 6 retained; eight ML algorithms tested; RF selected | PanNETs vs. pancreatic cancer | - RF ultrasomics signature: AUC: 0.99 training, 0.65 test<br>- Ultrasomics nomogram: AUC 1.00 training, 0.88 test<br>- Test accuracy: 0.80<br>-Test sensitivity: 0.73<br>- Test specificity: 0.85 | - Retrospective, single-center design<br>- Manual segmentation<br>- Heterogeneous EUS devices<br>- No external/prospective validation |
| Mo 2025 [24] | Retrospective, single-center EUS-based radiomics study; training/test cohorts; intratumoral and peritumoral manual ROI segmentation; no external or prospective validation | - Patients/cases: 257 patients divided into training and test cohorts<br>- Data units: conventional EUS images with intratumoral and 3-mm peritumoral ROIs; exact image count not reported | Intratumoral and 3-mm peritumoral radiomics; 107 features extracted; LASSO; six ML algorithms; SVM selected | PanNETs vs. pancreatic cancer | - Combined model: AUC 0.86 training, 0.82 test<br>- Test accuracy: 0.78<br>- Test sensitivity: 0.78<br>- Test specificity: 0.76<br>- Clinical-radiomic nomogram: AUC 0.90 | Retrospective single-center design; manual segmentation; conventional EUS only; long inclusion period; no external/prospective validation |

| | | | | | | |
|---|---|---|---|---|---|---|
| Mo 2024 [25] | Retrospective, single-center radiomics study; training/test cohorts; manual intratumoral and peritumoral ROI segmentation; no external or prospective validation | - Patients/cases: 106 PanNET patients, including training cohort of 74 and test cohort of 32<br>- Data units: conventional EUS images with intratumoral and 3-mm peritumoral ROIs; exact image count not reported | Intratumoral and 3-mm peritumoral radiomics; 107 features extracted; LASSO; six ML algorithms; "Light Gradient Boosting Machine" selected | Insulinomas vs. non-functional PanNETs | - Combined model: AUC 0.87 training, 0.83 test<br>- Test accuracy: 0.70<br>- Test sensitivity: 0.61<br>- Test specificity: 0.79<br>- Nomogram: AUC: 0.93 training, 0.91 test | - Retrospective, single-center design<br>- Small sample size<br>- Manual segmentation<br>- No external/prospective validation |
| Mo 2026 [26] | Retrospective single-center study; patient-level training/test split; standardized EUS images with radiomics and DL feature extraction; no external validation | - Patients/cases: 115 patients: 61 insulinomas and 54 non-functional PanNETs<br>- Data units: standardized EUS images used to extract 512 DL features and 107 radiomics features; exact image count not reported | 107 radiomics features and 512 DL features; LASSO feature selection; ExtraTrees-based radiomics and DL signatures; integrated DL-radiomics nomogram; Grad-CAM and SHAP for interpretability | Insulinomas vs. non-functional PanNETs | - ExtraTrees radiomics signature: AUC 0.90 in the training group and 0.85 in the test group; ExtraTrees DL signature: AUC 0.98 in the training group and 0.74 in the test group<br>- Integrated nomogram: AUC 0.98 in the training group and 0.84 in the test group | - Retrospective single-center design<br>- Small sample size<br>- Manual ROI segmentation<br>- Possible overfitting, especially for the DL signature<br>- Nomogram did not outperform the radiomics signature in the test cohort<br>- No external validation |
| Kiremitci 2025 [27] | Retrospective, single-center pilot study; image-level modeling with preprocessing, SMOTE augmentation, and 10-fold cross-validation; no patient-level modeling; no external validation | - Patients/cases: 44 patients<br>- Data units: 803 EUS images | CNN-based DL model applied to preprocessed EUS images; image normalization, thresholding, resizing, SMOTE augmentation, and 10-fold cross-validation | Prediction of PanNET grading from EUS images according to the 2017 WHO classification | - Overall sensitivity: 94.3%<br>- Overall specificity: 97.1%<br>- Overall PPV: 94.3%<br>- Overall NPV: 97.1%<br>- Overall accuracy: 96.2%<br>- Grade-specific accuracy: 93.1% for G1, 91.6% for G2, 98.0% for G3 | - Retrospective, single-center design<br>- Small cohort<br>- Image-level rather than patient-level modeling<br>- Marked class imbalance (only 5 G3 cases)<br>- Use of SMOTE-generated samples<br>- Heterogeneous FNA/FNB needles<br>- No external validation |
| Mo 2024 [28] | Retrospective, single-center EUS-based ultrasomics study; training and test cohorts; | - Patients/cases: 79 patients divided into training and test cohorts | 107 Ultrasomics features extracted from conventional EUS | Preoperative prediction of PanNET grading, | - AUC (XGBoost): 0.78-0.99<br>- Test accuracy: 0.78 | - Retrospective, single-center design<br>- Small cohort |

| | | | | | | |
|---|---|---|---|---|---|---|
| | manual ROI segmentation; no external validation | - Data units: conventional EUS images; 107 ultrasomics features extracted; exact image count not reported | images; LASSO feature selection (5 retained); comparison of eight machine-learning algorithms, with XGBoost selected as the optimal model; ultrasomics nomogram developed | distinguishing G1 from G2/G3 tumors | - Test sensitivity: 0.64<br>- Test specificity: 0.856<br>- Model outperforming EUS specialists in grading prediction accuracy | - Class imbalance (limited number of high-grade tumors)<br>- Manual ROI segmentation<br>- Heterogeneous EUS equipment<br>- No external validation |
| Mo 2025 [29] | Retrospective, single-center EUS-based ultrasomics study; training and test cohorts; intratumoral, peritumoral, and combined ROI analysis; manual ROI segmentation; no external prospective validation | - Patients/cases: 81 patients divided into training and test cohorts<br>- Data units: conventional EUS images with intratumoral, peritumoral, and combined ROIs; exact image count not reported | Ultrasomics features extracted from intratumoral, peritumoral, and combined ROIs on conventional EUS images; LASSO feature selection; multilayer perceptron models; SHAP-based interpretability | Preoperative prediction of PanNET grading, distinguishing G1 from G2/G3 tumors | - Best performance: combined intratumoral–peritumoral model<br>- AUC: 0.84-0.86<br>- Accuracy: 0.76<br>- Sensitivity: 0.70<br>- Specificity: 0.80<br>- PPV: 0.70<br>- NPV: 0.80 | - Retrospective, single-center design<br>- Small cohort<br>- Manual ROI segmentation<br>- Long study period<br>- No external prospective validation |
| Mo 2025 [30] | Retrospective, multicenter study; training cohort plus three external centers used as test cohort; manual ROI segmentation; no prospective validation | - Patients/cases: 108 patients, including training cohort and three external centers used as test cohort<br>- Data units: EUS images used for ResNet18-based DL feature extraction; exact image count not reported | Deep transfer learning using pre-trained ResNet18 to extract 512 DL features from EUS images; LASSO feature selection; comparison of multiple ML classifiers; Naive Bayes selected as optimal DL signature; integration with clinical/EUS features into a nomogram; Grad-CAM and SHAP for interpretability | Prediction of PanNET grading, distinguishing G1 from G2/G3 tumors | -AUC (Naive Bayes DL signature): 0.84-0.94<br>- AUC (final nomogram DL signature + lesion size/shape): 0.89-0.93 | - Retrospective design<br>- Modest external test cohort<br>- Manual ROI segmentation<br>- Heterogeneous EUS equipment and image acquisition parameters<br>- No prospective validation |

AI, artificial intelligence; EUS, endoscopic ultrasonography; PDAC, pancreatic ductal adenocarcinoma; SVM, support vector machine; DL, deep learning; DCGAN, deep convolutional generative adversarial network; AUC, area under the curve; PPV, positive predictive value; NPV, negative predictive value; YOLOv5m, You Only

Look Once version 5 medium; ROI, region of interest; R-CNN, region-based convolutional neural network; CLAHE, contrast-limited adaptive histogram equalization; ResNet, residual network; CNN, convolutional neural network; AIP, autoimmune pancreatitis; Grad-CAM, gradient-weighted class activation mapping; ML, machine learning; SHAP, SHapley additive exPlanations; PanNET, pancreatic neuroendocrine tumor; RF, random forest; LASSO, least absolute shrinkage and selection operator; SMOTE, synthetic minority over-sampling technique; FNA, fine-needle aspiration; FNB, fine-needle biopsy; XGBoost, extreme gradient boosting

**Table S4.** AI-assisted EUS and EUS-nCLE for differential diagnosis and risk stratification of pancreatic cystic lesions

| Study | Design and validation | Dataset | AI Method | Diagnostic Task | Main Findings | Limitations |
|---|---|---|---|---|---|---|
| Vilas-Boas 2022 [34] | Retrospective single-center pilot study using a prospectively maintained database; 80:20 image-level training/validation split; patient-level separation not reported; no external or prospective validation | - Patients/cases: 28 PCL: 17 mucinous and 11 non-mucinous<br>- Data units: 28 EUS videos; 5505 extracted frames, including 3725 from mucinous lesions and 1780 from non-mucinous cysts | Xception-based CNN with ImageNet transfer learning | Mucinous vs. non-mucinous PCL | - Accuracy: 98.5%<br>- Sensitivity: 98.3%<br>- Specificity: 98.9%<br>- PPV: 99.5%<br>- NPV: 96.4%<br>- AUC: 1.00<br>- Processing speed: 7.2 ms/frame | - Retrospective, single-center study<br>- Small patient cohort<br>- Single EUS platform<br>- Incomplete histologic confirmation<br>- No external/prospective validation |
| Seza 2025 [35] | Retrospective multicenter study; leave-one-out cross-validation after extensive image augmentation; no independent external or prospective validation | - Patients/cases: 49 surgically confirmed pancreatic cystic neoplasms: 25 SCN and 24 MCN<br>- Data units: four images per tumor for each modality: EUS, early-phase contrast-enhanced CT, T2-weighted MRI, and MRP; augmented to 39,200 images per modality | ResNet-based AI using single, double, triple, and four-modality combinations; sex included as clinical feature in selected models | SCN vs. MCN using EUS, CT, and MRI/MRP | - Four-modality AI: sensitivity 98.0%, specificity 100%, accuracy 99.0%; experienced experts using all modalities: sensitivity 78.0%, specificity 82.0%, accuracy 81.0% | - Retrospective design<br>- Small surgically selected cohort<br>- Extensive image augmentation<br>- Multimodal rather than EUS-only<br>- No independent prospective or external validation |
| Nguon 2021 [36] | Retrospective two-center study; patient-level repeated hold-out validation performed three times; manual ROI selection; training-data augmentation; no prospective or real-time validation | - Patients/cases: 109 patients: 60 MCN and 49 SCN<br>- Data units: 211 EUS images, with 1–4 images per patient; 130 MCN images and 81 SCN images | ResNet-50 CNN with transfer learning; single-ROI and multi-ROI testing; Grad-CAM | MCN vs. SCN | - Best single-ROI model: accuracy 82.8%, sensitivity 81.5%, specificity 84.4%, AUC 0.88<br>- Multi-ROI testing: accuracy 80.0%, sensitivity 76.0%, specificity 84.5%, AUC 0.84 | - Retrospective design<br>- Modest dataset<br>- Manual ROI selection<br>- Selected static images rather than real-time video analysis; no prospective validation. |
| Li 2026 [37] | Retrospective single-center EUS-feature–based ML study; patient-level 70:30 random training/test split; model tuning within the | - Patients/cases: 123 patients with indeterminate main pancreatic duct dilation: 83 CP and 40 MD-IPMN | Six ML algorithms; RF selected; ADASYN correction; calibration assessment; decision-curve analysis; SHAP | MD-IPMN vs. CP in patients with indeterminate MPD dilation on CT/MRI | - RF model: accuracy 0.917, F1-score 0.879, AUC 0.946; stone-negative subgroup: | - Retrospective, single-center design<br>- Modest sample size<br>- Differential reference standards |

| | | | | | | |
|---|---|---|---|---|---|---|
| | training set using 5-fold cross-validation; ADASYN applied only within training folds; final evaluation on an independent held-out test set; no external or prospective validation | - Data units: 21 predefined EUS features; no raw image/frame dataset | explainability; web-based risk interface | | F1-score 0.878, AUC 0.830<br>- SHAP identified ductal stones, diffuse lobularity, diffuse duct dilation, and atrophy as major predictors | - Possible verification/spectrum bias<br>- Feature-based model rather than direct image analysis<br>- External/prospective validation needed |
| Schulz 2023 [38] | Model-development study with independent test set including retrospective, prospective, and external cases; EUS image-based evaluation; no real-time validation | Patients/cases: 70 histologically proven IPMN patients: 43 training patients and 27 test patients, including 11 retrospective, 7 prospective, and 9 external test patients. Data units: 3355 EUS images for training and 1823 EUS images for testing | CNN with transfer learning | Histological risk stratification of IPMN: low-grade vs. high-grade/invasive carcinoma | - Test accuracy: 99.6%<br>- Accuracy of individual international consensus guidelines: 51.8%-70.4% | - Pilot study<br>- Small patient cohort<br>- Small prospective and external test subsets<br>- Image-based evaluation<br>- No real-time clinical validation |
| Krishna 2025 [39] | Post-hoc derivation analysis of prospectively enrolled EUS-nCLE studies from a single center; full-length unedited nCLE videos; cross-validated frame-level AI classification and case-level prediction; comparison with 16 blinded nCLE experts; no external or real-time AI deployment | - Patients/cases: 60 surgically confirmed BD-IPMNs, including 23 with HGD/IC; 16 blinded nCLE experts<br>- Data units: 60 full-length unedited EUS-nCLE videos; 260,117 nCLE frames analyzed; 52,523 structure-positive frames detected by nCLE-A | Two-step nCLE-AI model using automated structure detection and dysplasia classification; case-level logistic regression; combined analysis with revised Fukuoka criteria | Risk stratification of BD-IPMNs: LGD vs. HGD/IC | - nCLE-AI alone: sensitivity 87.0%, specificity 54.1%, accuracy 66.7%, AUC 0.70<br>- nCLE-AI + revised Fukuoka criteria: sensitivity 78.3%, specificity 78.4%, accuracy 78.3%, AUC 0.85<br>- Human observers + revised Fukuoka criteria: AUC 0.64 | - Single-center procedures<br>- Surgically selected cohort<br>- Post-hoc AI analysis<br>- No independent external validation<br>- Not yet evaluated as a real-time clinical tool |
| Machicado 2021 [40] | Post-hoc analysis of a single-center prospective EUS-nCLE study; CNN-CAD models applied to nCLE video frames; | Patients/cases: 35 histologically proven IPMNs, including 18 with HGD/adenocarcinoma | Two CNN-based CAD models: guided segmentation-based model assessing papillary epithelial | Risk stratification of IPMNs for high-grade dysplasia/adenocarcinoma | - CNN-CAD sensitivity 83.3% vs. 55.6% for AGA and Fukuoka guidelines | - Single-center post hoc analysis<br>- Small histologically confirmed cohort |

| | | | | | | |
|---|---|---|---|---|---|---|
| | comparison with AGA and revised Fukuoka guidelines; no prospective multicenter or real-time validation | - Data units: 15,027 EUS-nCLE video frames | features; holistic model using automated nCLE feature extraction | | - Accuracy 82.9%–85.7% vs. 68.6%–74.3% for guidelines; specificity 82.4%–88.2% vs. 82.4%–94.1% for guidelines | - Frame-based nCLE analysis<br>- No prospective multicenter validation<br>- Real-time implementation not established |

AI, Artificial Intelligence; EUS, endoscopic ultrasonography; EUS-nCLE, endoscopic ultrasound-guided needle-based confocal laser endomicroscopy; PCL, pancreatic cystic lesion; CNN, convolutional neural network; PPV, positive predictive value; NPV, negative predictive value; AUC, area under the curve; SCN, serous cystic neoplasm; MCN, mucinous cystic neoplasm; ResNet, residual network; CT, computed tomography; MRI, magnetic resonance imaging; MRP, magnetic resonance pancreatography; ROI, region of interest; Grad-CAM, gradient-weighted class activation mapping; ML, machine learning; CP, chronic pancreatitis; MD-IPMN, main-duct intraductal papillary mucinous neoplasm; RF, random forest; ADASYN, adaptive synthetic sampling; SHAP, SHapley additive exPlanations; MPD, main pancreatic duct; IPMN, intraductal papillary mucinous neoplasm; BD-IPMN, branch-duct intraductal papillary mucinous neoplasm; HGD/IC, high-grade dysplasia/invasive carcinoma; nCLE, needle-based confocal laser endomicroscopy; LGD, low-grade dysplasia; CAD, computer-assisted diagnosis; AGA, American Gastroenterological Association

**Table S5.** AI-assisted EUS for anatomical recognition, lesion segmentation, and procedural guidance in pancreaticobiliary EUS

| Study | Design and validation | Dataset | AI Method | Diagnostic Task | Main Findings | Limitations |
|---|---|---|---|---|---|---|
| Konikoff 2025 [41] | Single-center study using a prospectively collected and retrospectively analyzed dataset; stratified 70:15:15 training/validation/holdout test split; patient-level separation not explicitly reported; no external or prospective live validation | - Patients/cases: 165 cases<br>- Data units: 1497 EUS images, including 1034 training images, 260 validation images, and 203 test images | CADe segmentation framework; UperNet ConvNeXt and SegFormer models | Detection and segmentation of pancreatic abnormalities on EUS | - Best performance for SegFormer: accuracy 0.95, AUC 0.95, mean IoU 0.73, PPV 0.82, NPV 0.96, sensitivity 0.57, specificity 0.98 | - Single-center study<br>- Single EUS device<br>- No direct comparison with human readers<br>- Segmentation ground truth based on expert annotations<br>- No prospective live clinical validation |
| Zhang 2025 [42] | Retrospective multicenter static-image model-development and validation study; internal test set, external validation set, and man–machine competition test set; no prospective real-time EUS evaluation | - Patients/cases: 1152 training patients; 136 internal-test cases; 108 external-validation patients.<br>- Data units: 45,737 training images, 2881 internal-test images, 2747 external-validation images; 340 external-validation images used for man–machine comparison | EUS-AI system combining Mean Teacher for eight-station recognition + U-Net v2 for pancreas/biliopancreatic duct segmentation | Biliopancreatic EUS station recognition + anatomical segmentation | - Mean Teacher station-recognition accuracy: 95.6% internal vs. 92.7% external<br>- AI outperformed junior/mid-level endoscopists<br>- Station classification accuracy: 97.9% vs. 81.9%<br>- Pancreas IoU: 0.67 vs. 0.62<br>- Biliopancreatic duct IoU: 0.57 vs. 0.51 | - Retrospective design<br>- Static-image study<br>- Limited interpretability<br>- Limited to two EUS platforms<br>- Need for broader validation<br>- No real-time EUS evaluation |
| Li 2024 [43] | Retrospective single-institution model-development study; patient-level random training/validation/test split at 3:1:1; semi-supervised learning using additional unlabeled EUS images; no external, prospective, or real-time clinical validation | - Patients/cases: 499 patients from four categories: normal pancreas, pancreatic tumors, chronic pancreatitis, and AIP<br>- Data units: 9555 pathologically proven labeled EUS images and 15,500 unlabeled EUS images; training set 300 patients/5712 images, validation set 99 | SSM-Net with saliency-aware contrastive learning, U-Net-based segmentation, channel attention blocks, merged global attention module, and feature similarity loss | Joint pancreatic lesion segmentation and classification on EUS images | - Test set: DSC 0.90, mIoU 0.74, classification accuracy 0.79, recall 0.61, precision 0.55, F1-score 0.58<br>- Outperforming compared semi-supervised and multi-task methods | - Retrospective design<br>- Single-institution dataset<br>- Classification grouped heterogeneous pancreatic tumors together<br>- Model complexity may limit clinical translation<br>- No prospective, external, or real-time clinical validation |

| | | | | | | |
|---|---|---|---|---|---|---|
| | | patients/1732 images, test set 100 patients/2111 images | | | | |
| Seo 2022 [44] | Retrospective, single-center study; 5-fold cross-validation; each fold included 66 images from 30 patients; no external, prospective, or real-time validation | - Patients/cases: 150 patients with pancreatic cancer<br>- Data units: 330 linear EUS images; 5-fold cross-validation with 66 images from 30 patients per fold | - DAF-Net segmentation model with deep attention features<br>- Comparison with U-Net and Attention U-Net | Semantic segmentation of pancreatic cancer on EUS images | - DSC: 82.8%<br>- IoU: 72.3%<br>- AUC: 0.93<br>- Sensitivity: 89.0%<br>- Specificity: 98.1%<br>- Precision: 85.1% | - Retrospective, single-center design<br>- Limited image dataset<br>- Linear EUS images only<br>- No external or prospective validation<br>- No real-time workflow assessment |
| Oh 2022 [45] | Retrospective, two-dataset study; 5-fold cross-validation on training data; internal test and external test; additional cross-over analysis training on Dataset B and testing on Dataset A; no prospective or real-time validation | - Patients/cases: Dataset A, 52 patients; Dataset B, 59 patients<br>- Data units: Dataset A, 57 EUS images (43 training images + 14 internal test images); Dataset B, 364 EUS images used as external test set; cross-over analysis used 288 training images and 76 internal test images from Dataset B, with Dataset A as external test set | U-Net-based models: Basic U-Net, Residual U-Net, U-Net++, Attention U-Net | Automatic segmentation of PCLs on EUS images | - Best internal performance for Attention U-Net: DSC 0.80, IoU 0.74<br>- Best external performance for Basic U-Net: DSC 0.70, IoU 0.59 | - Retrospective design<br>- Modest sample size<br>- Operator/device-dependent variability;<br>- No real-time validation |
| Iwasa 2021 [46] | Retrospective, two-center CE-EUS video-frame study; 4-fold cross-validation; manual B-mode tumor annotation used as ground truth; segmentation-only analysis; no external or prospective real-time clinical validation | - Patients/cases: 100 patients with pancreatic solid tumors<br>- Data units: 90-second CE-EUS videos converted from 30 to 6 frames/s; manual B-mode tumor annotations used as ground truth | U-Net segmentation model; manual B-mode tumor annotation as ground truth; 100 epochs; 4-fold cross-validation | - Automatic segmentation of pancreatic tumors on CE-EUS video images | - Median IoU: 0.77<br>- Segmentation better with clear tumor boundaries: IoU Tumor Boundary-1 0.80 vs. Tumor Boundary-3 0.68 ($p < 0.01$)<br>- Respiratory movement not significantly affecting IoU<br>- IoU was lower when tumor boundaries were unclear | - Retrospective, two-center design<br>- Modest sample size<br>- No lesion classification<br>- Only U-Net was tested |

| | | | | | | |
|---|---|---|---|---|---|---|
| Bang 2026 [47] | Prospective single-center nonrandomized real-time comparative clinical validation study; concurrent blinded assessment by expert conventional EUS and AI-EUS; separate model-training and performance-testing phases; no external multicenter validation | - Patients/cases: 510 patients overall: 150 for model training, 52 for performance testing, and 308 for prospective clinical validation<br>- Data units: 42,777 EUS frames overall, including 29,586 training frames, 3127 performance-testing frames, and 10,064 clinical-validation frames | PANCRAIEUS real-time AI-EUS system using Mask R-CNN + YOLOv8; trained/tested on 32,713 EUS frames from 202 patients | Real-time detection and segmentation of solid pancreatic masses | - AI-EUS solid mass detection: 97.1% vs. 100% with expert conventional EUS<br>- Sensitivity: 97.1%<br>- Specificity: 100%<br>- Accuracy: 98.5%<br>- Detection by size: ≤ 15 mm, 81.8%; 16–29 mm, 100%; ≥ 30 mm, 98.5% | - Single-center study<br>- Single EUS processor<br>- External multicenter and multi-platform validation needed |
| Tang 2023 [48] | Retrospective multicenter model-development phase with 8:2 training/testing split, followed by a single-center randomized clinical phase evaluating CE-EUS MASTER-guided EUS-FNA; no external prospective clinical validation | - Patients/cases: Model-development phase included 296 CE-EUS videos from 296 patients for TIC-based classification; clinical phase included 39 randomized patients<br>- Data units: 4342 CE-EUS images for segmentation, including 3546 pancreatic cancer images and 886 benign pancreatic mass images; 296 CE-EUS videos for TIC-based classification | CE-EUS MASTER: UNet++/ResNet-50 segmentation + Random Forest classification using time–intensity curve features; real-time thermogram for EUS-FNA guidance | - Benign vs. malignant pancreatic mass<br>- AI-guided EUS-FNA targeting | - Model 1: mean IoU 0.708, accuracy 87.8%<br>- Model 2: accuracy 88.9%, sensitivity 100%, specificity 75.0%, PPV 83.3%, NPV 100%<br>- CE-EUS MASTER-guided EUS-FNA: accuracy 93.8%; sensitivity 90.9%, specificity 100%, PPV 100%, NPV 83.3%; first-pass malignant diagnosis 80.0% vs. 33.3% | - Small single-center cohort<br>- Mainly focused on pancreatic cancer vs. mass-forming pancreatitis<br>- Larger multicenter validation needed |
| Tang 2023 [49] | Retrospective single-center model-development study with 8:2 image-level training/testing split; crossover trainee-training study based on video replay; no prospective clinical validation | - Patients/cases: 270 patients/videos for model development; 50 patients and 8 EUS trainees in the training study<br>- Data units: 4530 EUS images from 270 videos, including 3624 training images and 906 testing | CE-EUS MASTER real-time capture and segmentation system based on UNet++ with ResNet-50 backbone; expert manual annotation as reference standard | - Real-time capture and segmentation of solid pancreatic masses<br>- AI-assisted EUS trainee training | - Test set: median IoU 0.73, DSC 0.76, recall 0.94, precision 0.64, accuracy 0.84<br>- AI-assisted learning increased mean IoU from 0.80 to 0.87 and reduced lesion-identification time | - Retrospective, single-center design<br>- Relatively small training experiment<br>- Based on video replay rather than real-time procedures<br>- No prospective clinical validation |

| | | | | | | |
|---|---|---|---|---|---|---|
| | | images; training study used 100 videos from 50 patients | | | - Lesion-identification time decreased from 22.75 to 17.98 s for body/tail lesions and from 34.21 to 25.92 s for head/uncinate lesions | |

AI, Artificial Intelligence; EUS, Endoscopic Ultrasonography; CADe, Computer-Aided Detection; AUC, Area under the Curve; IoU, Intersection over Union; PPV, Positive Predictive Value; NPV, Negative Predictive Value; EUS-AI, EUS artificial intelligence; U-Net, U-shaped convolutional neural network; SSM-Net, semi-supervised multi-task network; DSC, Dice similarity coefficient; mIoU, mean intersection over union; DAF-Net, deep attention feature network; PCL, pancreatic cystic lesion; CE-EUS, contrast-enhanced endoscopic ultrasonography; PANCRAIEUS, pancreatic artificial intelligence-enhanced endoscopic ultrasonography; Mask R-CNN, mask region-based convolutional neural network; YOLOv8, You Only Look Once version 8; TIC, time–intensity curve; FNA, fine-needle aspiration; ResNet, residual network

**Table S6.** AI-based cytology tools intended to support ROSE, specimen adequacy assessment, and histopathology using pancreatic EUS-FNA/FNB-derived material

| Study | Design and validation | Dataset | AI Method | Diagnostic Task | Main Findings | Limitations |
|---|---|---|---|---|---|---|
| Zhang 2022 [57] | Retrospective, multicenter diagnostic study; patient-independent training, validation, internal testing, and external testing datasets; per-image and per-patient cancer-identification analyses; human–machine comparison; no prospective real-time validation | - Patients/cases: 194 patients from four institutions<br>- Data units: 5345 cytopathological slide images: 2166 training images, 695 validation images, 1162 internal-testing images, and 1322 external-testing images; human–machine comparison datasets selected from internal and external testing sets | U-Net-based DCNN with ResNet101 encoder for segmentation of stained cell clusters and cancer cell clusters | - AI-based cytology support intended for ROSE<br>- Assessment of sample adequacy + identification of PDAC cell clusters | - F1-score (cell-cluster segmentation): 0.90-0.94<br>- AUC (image-level): 0.95-0.98<br>- AUC (patient-level) 0.98-1.00<br>- System outperforming trained endoscopists + comparable to cytopathologists | - Retrospective design<br>- Selected high-quality cytopathological images<br>- Retrospective external testing only<br>- No prospective real-time validation |
| Fang 2025 [58] | Retrospective, multicenter cytology-image study; images from one center randomly divided into training, validation, and internal testing sets, with another center used as external testing set; separate cytopathologist–system competition and "atypical" analysis; patient-level separation not clearly reported; no prospective validation | - Patients/cases: 210 patients with pancreatic masses from four hospitals<br>- Data units: 3213 cytological images acquired at unified magnification; 60 images used for cytopathologist–system comparison; 33 "atypical" images analyzed separately | Semi-supervised CNN system using U-Net with ResNet50 encoder; teacher–student self-training | - PDAC vs. non-PDAC classification and segmentation<br>- Evaluation of "atypical" cases | - Segmentation F1-score: 93.5-93.7%<br>- PDAC classification: accuracy 93.9%-95.1% across internal and external testing; AUC 0.97-0.99<br>- In human–machine comparison: accuracy 90.0%, with high agreement with senior cytopathologists<br>- Accuracy for "atypical" cases: 78.8% | - Retrospective design<br>- Binary PDAC vs. non-PDAC classification only<br>- "Atypical" analysis based on a small image set<br>- No prospective multicenter validation |
| Lin 2023 [59] | Proof-of-concept study; single-center model development with internal validation and small independent image-level test set; tiled-image analysis; no prospective validation | - Patients/cases: 51 patients overall; 42 patients for model development/internal validation and 9 patients for external testing<br>- Data units: 1160 digitized EUS-FNA slide images; | ResNet101V2-based ROSE-AI model trained on tiled cytology images for | - AI-based digital cytology support intended for ROSE during EUS-FNA<br>- Detection of cancer cells on digitized cytology slides | - Accuracy: 83.4%-88.7%<br>- Sensitivity: 78.0%<br>- Specificity: 90.6%<br>- PPV: 60.7%<br>- NPV: 95.7% | - Proof-of-concept design<br>- Small cohort<br>- Category imbalance<br>- Limited independent test set |

| | | | | | | |
|---|---|---|---|---|---|---|
| | | model development used 467 images, including 367 training images/3642 tiles and 100 internal-validation images/916 tiles; external test included 693 images | cancer-cell detection | | | - No prospective validation |
| Tian 2025 [60] | Retrospective single-center mobile-phone cytology-image study; two-stage model with tissue-cell detection followed by malignant-cell classification; separate whole-image test set; patient-level separation not clearly reported; no external or prospective validation | - Patients/cases: 92 patients with pancreatic solid lesions<br>- Data units: 882 mobile phone-captured EUS-FNA cytology images; first-stage tissue-cell dataset included 41 fully annotated images/2487 cells; second-stage classification used training blocks from 329 fully labeled and 439 incompletely labeled images, validation blocks from 54 fully labeled images, and 60 separate completely labeled whole images for testing | Two-stage DL model combining YOLOv8n-p2 for tissue-cell detection + DenseNet201 | - AI-based cytology support intended for ROSE of EUS-FNA specimens<br>- Whole-image malignancy classification | Separate whole-image test set: accuracy 93.3%, sensitivity 93.3%, specificity 93.3%, PPV 93.3%, NPV 93.3%, F1-score 93.3%, AUC 0.96 | - Retrospective, single-center design<br>- Modest patient cohort<br>- Mobile phone image variability<br>- Partial annotation of some training images<br>- No prospective external validation |
| Fujii 2024 [61] | Retrospective single-center study with five-fold cross-validation; divided-image/tile-based analysis after digitization of EUS-FNA slides; no external validation with independent new images or patients | - Patients/cases: 45 patients: 36 pancreatic cancer and 9 non-pancreatic cancer<br>- Data units: 70 Diff-Quik-stained EUS-FNA slides; 4059 divided images, including 2079 with cancer cells and 2080 without cancer cells | Transformer-based ROSE-AI system using Vision Transformer; comparison of data-augmentation strategies including geometric transformation, color-space | - AI-based cytology support intended for ROSE of EUS-FNA specimens<br>- Cancer-cell detection<br>- Evaluation of data augmentation | - Without data augmentation: sensitivity 87.5%, specificity 79.7%, accuracy 83.7%, AUC 0.90<br>- Best performance for Geometric transformation: sensitivity 91.4%, specificity 85.0%, accuracy 88.2%, AUC 0.95 | - Retrospective, single-center design<br>- No external validation with new images<br>- Five-fold cross-validation only<br>- Limited disease spectrum |

| | | | | | | |
|---|---|---|---|---|---|---|
| | | | transformation, kernel filtering, and combinations | | | |
| Ashida 2026 [62] | Retrospective single-center study; patient-level random split into training, validation, and independent test cohorts, with no patient data split between cohorts; human-reader comparison using test-cohort cell clusters; no prospective multicenter validation | - Patients/cases: 137 patients: 96 training, 15 validation, and 26 test patients; 21 endosonographers and 5 cytotechnologists in the comparison study<br>- Data units: 5157 training, 615 validation, and 2521 test cell-cluster images; 1,097,840 training, 31,817 validation, and 30,252 test tiled images; 120 test-cohort cell clusters used for human-reader comparison | Semantic segmentation model with transformer-based encoder and U-Net decoder; annotated cell clusters classified into five cytological classes and grouped into three or two diagnostic categories | - Rapid AI-based cytologic classification intended to support ROSE during EUS-FNA<br>- Cytologic classification of pancreatic cell clusters as benign vs. malignant + three-category classification | - Three-category classification accuracy: 89.8%<br>- For benign vs. malignant classification in the test cohort: sensitivity 89.3%, specificity 98.1%, accuracy 95.1%<br>- In the 120-cell-cluster comparison cohort: accuracy 93.3%, evaluation time 6.04 s (supporting rapid post-acquisition inference but not yet proving prospectively validated live AI-ROSE) | - Retrospective, single-center design<br>- Limited variety of pancreatic tumor types<br>- Lower performance for class 3/atypical clusters<br>- Possible image-selection bias<br>- No prospective multicenter validation |
| Qin 2023 [63] | Retrospective single-center HSI/RGB cytology study; patient-level 60:20:20 training/validation/test split with no patient overlap; additional temporally later test set; no multicenter validation | - Patients/cases: 72 histologically confirmed cases: 39 PDAC and 33 benign pancreatic cases; 60 cases used for training/validation/testing and 12 cases used as additional test set<br>- Data units: 1913 paired HSI and RGB images from EUS-FNA liquid-based cytology slides | ResNet18-HSI-SimSiam DL model using hyperspectral imaging vs. RGB-based CNN model; AGF-Visualization for explainability | PDAC vs. benign pancreatic cells | - Final ResNet18-HSI-SimSiam model outperformed the RGB-based model: accuracy 92.0%, sensitivity 93.1%, specificity 91.2%, AUC 0.96<br>- Additional test set: accuracy 92.3%, AUC 0.97<br>- Model activation mainly driven by nuclear features | - Retrospective, single-center design<br>- Small cohort<br>- Limited disease spectrum<br>- No multicenter validation |
| Qin 2026 [64] | Retrospective single-center HSI/RGB cytology study; training, validation, internal test, and temporally separate | - Patients/cases: 59 patients: 35 PDAC and 24 PanNETs; 29 training, 10 validation, 10 internal-test, and 10 | Modified ResNet18 CNN using hyperspectral | PDAC vs. PanNET | - HSI-based ResNet18 outperformed RGB-based analysis | - Retrospective, single-center design<br>- Small cohort |

| | | | | | | |
|---|---|---|---|---|---|---|
| | test set reported by the authors as external testing; no multicenter validation | external/temporal-test patients<br>- Data units: 2014 paired HSI and RGB images from EUS-FNA liquid-based cytology slides | imaging vs. RGB-based analysis; AGF-Visualization for explainability | | - Test set 1: accuracy 92.8%, sensitivity 90.8%, specificity 94.7%, AUC 0.97<br>- External/temporal test set: accuracy 95.4%, sensitivity 96.9%, specificity 92.4%, AUC 0.99<br>- Model activation mainly driven by nuclear features | - Limited disease spectrum<br>- No multicenter validation |
| Ishikawa 2022 [65] | Retrospective single-center EUS-FNB specimen-adequacy study; initial AlexNet-based deep learning analysis with 3-fold cross-validation; expanded contrastive-learning analysis linking stereomicroscopic and H&E-stained images; specimen-level adequacy assessment; no external or prospective validation | - Patients/cases: initial DL analysis included 63 patients; expanded contrastive-learning analysis included 96 patients<br>- Data units: 98 fresh EUS-FNB specimens in the initial analysis and 173 specimens in the expanded analysis; stereomicroscopic images of fresh 22G Franseen EUS-FNB samples, linked to H&E-stained images and core-tissue area for contrastive learning | Initial AlexNet-based DL model using stereomicroscopic images; subsequent contrastive learning model linking stereomicroscopic images with H&E-stained images and marked core-tissue areas | - Predicting adequacy of fresh EUS-FNB specimens for histological diagnosis<br>- AI-based alternative to MOSE | - Accuracy: 71.8% for AlexNet vs. 81.6% for MOSE<br>- With contrastive learning, AI performance comparable to MOSE: accuracy 84.4%, sensitivity 90.3%, specificity 53.5%, PPV 90.9%, and NPV 51.7%<br>- Adequate histological diagnosis for larger tissue area (2.12 vs 0.46 mm²) | - Retrospective, single-center design<br>- Small cohort<br>- AI assessed specimen adequacy rather than final disease diagnosis<br>- No prospective or external validation |
| Naito 2021 [56] | Retrospective single-center EUS-FNB histopathology WSI study; training, validation, and test split; test set restricted to WSIs with complete diagnostic agreement among three pancreatic pathologists; no external validation | - Patients/cases: patient number not separately reported<br>- Data units: 532 EUS-FNB histopathological WSIs: 372 training WSIs, 40 validation WSIs, and 120 test WSIs; 62 diagnostically discordant WSIs were excluded from the test set | EfficientNet-B1 CNN trained using transfer learning and fully supervised learning on annotated EUS-FNB histopathological WSIs | - Detection of PDAC on EUS-FNB histopathological WSI | - AUC: 0.98<br>- Accuracy: 0.94<br>- Sensitivity: 0.93<br>- Specificity: 0.97<br>- F1-score: 0.96<br>- Ability to detect isolated carcinoma components in blood- and fibrin-rich samples | - Retrospective, single-center design<br>- Modest test set<br>- Exclusion of diagnostically discordant cases<br>- No external validation |

| | | | | | | |
|---|---|---|---|---|---|---|
| Udriștoiu 2024 [66] | Retrospective two-center WSI segmentation study; separate training, validation, and test sets within each dataset; cross-dataset testing between the two centers; patch-level segmentation; no prospective validation | - Patients/cases: patient number not separately reported; two EUS-FNB PDAC WSI datasets from two medical centers<br>- Data units: 64 WSIs and 8334 patches overall. Dataset 1: 31 WSIs/4040 patches, including 2228 training, 712 validation, and 1100 test patches. Dataset 2: 33 WSIs/4294 patches, including 2294 training, 800 validation, and 1200 test patches | Comparison of seven U-Net-based segmentation architectures: Vanilla U-Net, Attention U-Net, Dense U-Net, Inception U-Net, U-Net++, Residual U-Net, and SE-U-Net | - Automatic segmentation/localization of PDAC tissue | - Best performance for Inception U-Net: DSC 97.8% and IoU 0.87 in Dataset 1; DSC 95.7% and IoU 0.79 in Dataset 2<br>- Cross-dataset testing: DSC 93.1%/92.1% and IoU 0.74/0.81 | - Retrospective design<br>- Modest number of WSIs<br>- Heterogeneous equipment<br>- Need for larger multicenter validation and consensus expert annotations |

Studies summarized in this table refer to AI applications using pancreatic specimens obtained through EUS-FNA or EUS-FNB. Broader digital cytology or pathology literature is discussed only as methodological background and is not presented as direct evidence of AI performance in pancreatic EUS.

AI, artificial intelligence; ROSE, rapid on-site evaluation; EUS, endoscopic ultrasonography; FNA, fine-needle aspiration; FNB, fine-needle biopsy; U-Net, U-shaped convolutional neural network; DCNN, deep convolutional neural network; ResNet, residual network; PDAC, pancreatic ductal adenocarcinoma; AUC, area under the curve; CNN, convolutional neural network; PPV, positive predictive value; NPV, negative predictive value; DL, deep learning; YOLOv8n-p2, You Only Look Once version 8 nano-p2; DenseNet201, densely connected convolutional network 201; HSI, hyperspectral imaging; RGB, red, green, blue; AGF, attribution-guided factorization visualization; PanNET, pancreatic neuroendocrine tumor; H&E, hematoxylin and eosin; MOSE, macroscopic on-site evaluation; WSI, whole-slide imaging; SE-U-Net, squeeze-and-excitation U-Net; DSC, Dice similarity coefficient; IoU, intersection over union
